# Metabolic Syndrome: From Mechanisms to Therapeutic Interventions

**DOI:** 10.1002/mco2.70934

**Published:** 2026-08-24

**Authors:** Mengyao Yan, Qian Xiang

**Affiliations:** ^1^ Institute of Clinical Pharmacology Peking University First Hospital Beijing China; ^2^ Department of Pharmacy Administration and Clinical Pharmacy School of Pharmaceutical Sciences Peking University Health Science Center Beijing China

**Keywords:** adipose tissue, gut microbiota, gut microbiota‐derived metabolites, metabolic syndrome

## Abstract

Metabolic syndrome describes a set of risk factors that can eventually lead to the occurrence of cardiovascular and cerebrovascular disease. Metabolic syndrome has emerged as a significant global health issue, associated with various metabolic diseases, including obesity, diabetes, hypertension, dyslipidemia, chronic kidney disease, metabolic dysfunction‐associated steatotic liver disease, and other metabolic disorders. Here, we summarize the intricate mechanisms including insulin resistance, chronic low‐grade inflammation, oxidative stress, and epigenetic modifications, and how they contribute to the disease progression of metabolic syndrome. The gut–adipose tissue axis in the progression of metabolic syndrome is emphasized here, mainly involving adipocyte‐derived extracellular vesicles and adipokines, as well as specific gut microbiota and their secreted factors, such as lipopolysaccharide, short‐chain fatty acids, endocannabinoids, bile acids, aryl hydrocarbon receptor ligands, and tryptophan derivatives. Furthermore, the contemporary management for metabolic syndrome mainly includes some established pharmacological treatments such as GLP‐1 receptor agonists, SGLT2 inhibitors, and RAAS inhibitors, as well as promising emerging therapies targeting the gut–adipose tissue axis such as lifestyle modifications, prebiotics, probiotics, synbiotic supplements, FMT, bariatric surgery, CB1R antagonists, and novel pharmacological agents. These strategies may pave the way for the development of effective treatments for metabolic diseases in future research.

## Graphical abstract

A comprehensive overview of molecular mechanisms delves into the potential disease mechanisms of metabolic syndrome, including complex mechanisms such as insulin resistance, chronic low‐grade inflammation, oxidative stress, and epigenetic modifications, and how these mechanisms contribute to the development of metabolic syndrome. Here, the relationship between the gut–adipose tissue axis and the development of metabolic syndrome is emphasized. The complex interactions involved in the metabolic syndrome of gut–adipose tissue axis include the effects of extracellular vesicles derived from adipocytes and adipokines, as well as specific gut microbiota and their secreted factors. Potential interventions for the progress of metabolic syndrome mainly include some specific drugs for Type 2 diabetes, hypertension, dyslipidemia, and chronic kidney disease, as well as some drugs for gut–adipose tissue axis, mainly including lifestyle modifications, prebiotics, probiotics, synbiotic supplements, FMT, bariatric surgery, CB1R antagonists, and novel pharmacological agents.

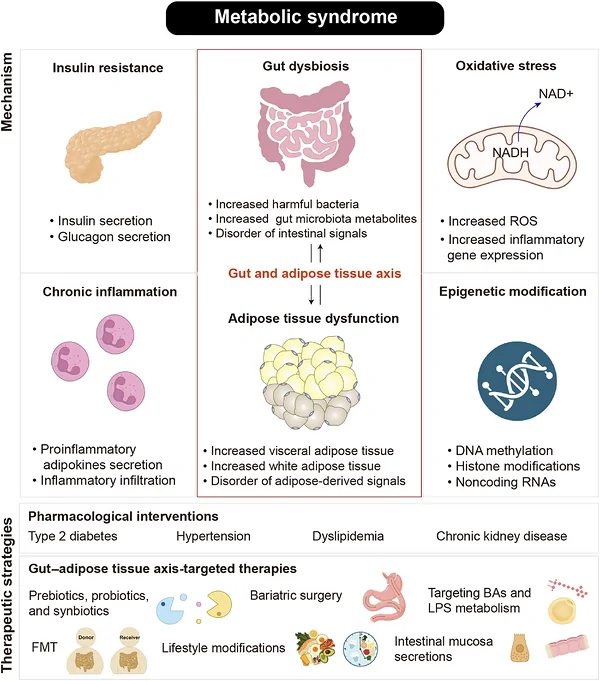

## 1 Introduction

Metabolic syndrome is a cluster of metabolic disorders characterized by central obesity, insulin resistance, dyslipidemia, and impaired glucose homeostasis. It has emerged as a significant global health issue, associated with various metabolic diseases, including obesity, diabetes, hypertension, dyslipidemia, chronic kidney disease, inflammatory bowel disease (IBD), and metabolic dysfunction‐associated steatotic liver disease (MASLD) [1, 2, 3]. Despite its increasing prevalence, the pathophysiological mechanisms underlying metabolic syndrome remain incompletely understood, with ongoing multidimensional debates regarding its core driving factors within the academic community.

Insulin resistance has long been considered a central initiating event in metabolic syndrome. However, accumulating evidence suggests that it is more likely the consequence of multiple upstream pathological processes acting in concert rather than a singular primary defect [4]. Chronic low‐grade inflammation, mitochondrial energy metabolism disorders, endoplasmic reticulum stress, redox imbalance, and disturbances in gut microbiota‐host cometabolism have been shown to independently or synergistically impair insulin signaling and promote systemic metabolic dysregulation [5]. Of particular significance is the evolving conceptualization of obesity itself: adiposity is not intrinsically pathogenic—rather, its metabolic consequences are critically determined by the functional status of adipose tissue (AT). Abnormal deposition of visceral and ectopic fat (in the liver, myocardium, and skeletal muscle) typically coincides with adipocyte hypertrophy, tissue hypoxia, fibrosis, and immune cell infiltration, collectively driving enhanced lipolysis, free fatty acid overflow, and increased release of proinflammatory cytokines, such as tumor necrosis factor‐α (TNF‐α) and interleukin (IL)‐6, and lipotoxic molecules [6]. Consequently, the research paradigm is undergoing a fundamental shift from an “obesity‐centered theory” to an “adipose tissue dysfunction‐centered theory,” emphasizing that metabolic syndrome is essentially an organ‐to‐organ communication disorder driven by multiple factors [7].

The gut is an active endocrine organ in the human body that regulates key physiological processes, including glucose and lipid metabolism, appetite, and energy balance, by emitting signaling molecules [8, 9]. Disruption of the gut microbiota can lead to various pathological conditions, such as chronic low‐grade inflammation, which damages the mucosal barrier, causes systemic bacterial translocation, and activates inflammatory processes [10]. AT is an active, multifunctional metabolic organ and a crucial component of the human body. It is primarily composed of mature adipocytes, preadipocytes, and immune cells and is regulated by blood vessels and nerves [11, 12]. There are two widely accepted classification systems for AT: one based on location—subcutaneous and visceral AT—and the other based on morphology and function—white adipose tissue (WAT) and brown adipose tissue (BAT). WAT is primarily responsible for energy storage [13]. In contrast, BAT possesses unique capabilities for energy dissipation, particularly through thermogenesis. BAT is predominantly found in areas such as the neck and around major blood vessels and is more metabolically active [14]. Furthermore, the amount of human BAT is highly influenced by age and environmental factors. A significant reduction in BAT activity in individuals with obesity or advanced age suggests a role for BAT in the metabolic dysfunction observed in both humans and mice [15, 16]. Importantly, an increasing number of studies indicate that adipocyte‐derived extracellular vesicles (ADEVs) and adipokines affect the gut microbiota, potentially disrupting nutrient and metabolic sensing and leading to miscommunication between AT and the gut. This constitutes a key driver of the metabolic complications clustered in metabolic dysfunction [17]. Furthermore, disruption of the gut barrier affects the release of gut hormones. For instance, gut dysbiosis can lead to decreased secretion of glucagon‐like peptide‐1 (GLP‐1), which regulates the metabolism and function of both WAT and BAT in MASLD [8, 18, 19]. This suggests a bidirectional pathway between the gut microbiota and AT.

This review summarizes the molecular mechanisms of metabolic syndrome, with particular emphasis on the gut–AT axis as an integrative framework linking gut dysbiosis, adipose dysfunction, microbial metabolites, systemic inflammation, and metabolic complications. We further discuss therapeutic strategies targeting this axis and aim to connect mechanistic insights with clinical translation.

## 2 Molecular Mechanisms Underlying Metabolic Syndrome

**FIGURE 1:**
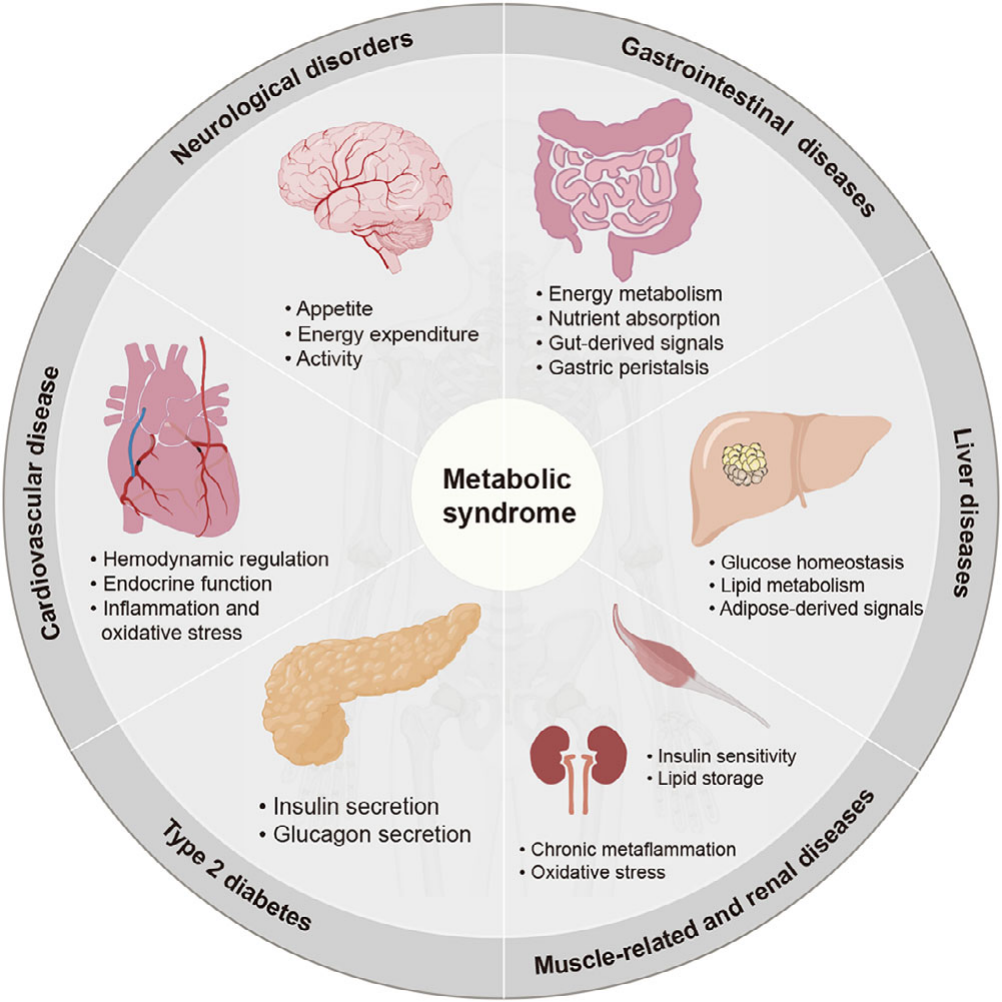
Metabolic syndrome in different metabolic organs and diseases. Metabolic syndrome involves multiple organs and is associated with several diseases, including Type 2 diabetes, cardiovascular disease, neurological disorders, gastrointestinal diseases, liver diseases, muscle‐related diseases, and renal disease.

**FIGURE 2:**
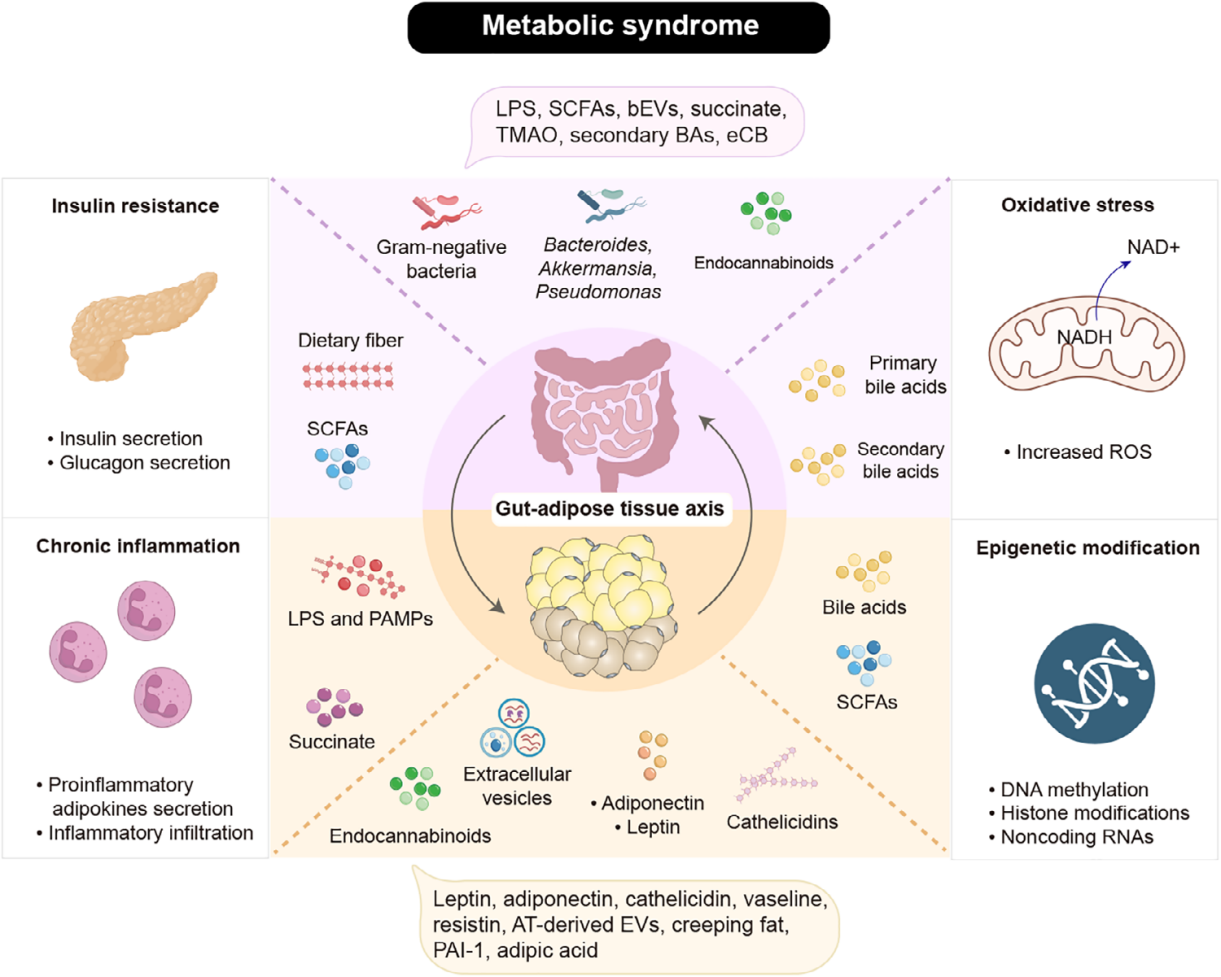
The potential pathogenesis of metabolic syndrome. The intricate mechanisms include insulin resistance, chronic low‐grade inflammation, oxidative stress, epigenetic modifications, intestinal disorder, and adipose tissue dysfunction. Importantly, there are bidirectional communication pathways between the gut and adipose tissue.

Metabolic syndrome is a systemic, multifactorial disorder that significantly increases the risk of numerous chronic diseases. It is not merely a high‐risk systemic metabolic state with extensive pathophysiological implications. Clinically, metabolic syndrome is strongly associated with the development and progression of Type 2 diabetes, MASLD, cardiovascular disease, chronic kidney disease, neurological disorders, and muscle‐related diseases [20] (Figure 1). The pathogenesis of metabolic syndrome involves a vicious cycle of interconnected conditions, including insulin resistance, chronic low‐grade inflammation, oxidative stress, and environmentally induced epigenetic changes, which mutually reinforce one another (Figure 2).

### 2.1 The Gut–AT Axis as the Pathogenic Hub

The gut, or gastrointestinal tract, is considered one of the body's primary endocrine organs. The gut microbiota refers to the collective community of symbiotic microorganisms residing within the gastrointestinal tract. These microorganisms are vast in number, reaching trillions of cells, and encompass various types of organisms, including bacteria, fungi, archaea, and viruses. They constitute a key and complex component of healthy physiological functions [21]. In the adult gut, over 90% of microorganisms belong to two main bacterial phyla: *Bacteroidetes* and *Firmicutes*, while other phyla such as *Actinobacteria*, *Cyanobacteria*, *Fusobacteria*, *Proteobacteria*, and *Verrucomicrobia* are present in relatively smaller numbers [22, 23].

The bidirectional relationship between the gut and AT may contribute to metabolic syndrome and its related complications, including obesity, diabetes, hypertension, dyslipidemia, chronic kidney disease, IBD, and MASLD. Numerous animal studies have demonstrated that adipocytes release adipose‐derived factors—mainly adipokines—as well as extracellular vesicles, which influence gut microbiota and intestinal function [24, 25]. For example, adipocyte‐selective ablation of a disintegrin and metalloproteinase domain 17 prevents the effects of fecal microbiota transplantation (FMT) from nonresponders on the elevation of soluble IL‐6 receptor, thereby restoring the efficacy of exercise‐modulated gut microbiome in counteracting glucose intolerance and insulin resistance in obese mice [26]. Additionally, another animal study shows that adiponectin regulates Crohn's disease through the AdipoR1/2‐metabolic pathway and ulcerative colitis through the AdipoR1–inflammation axis or the AdipoR2–fibrosis pathway [27].

**FIGURE 3:**
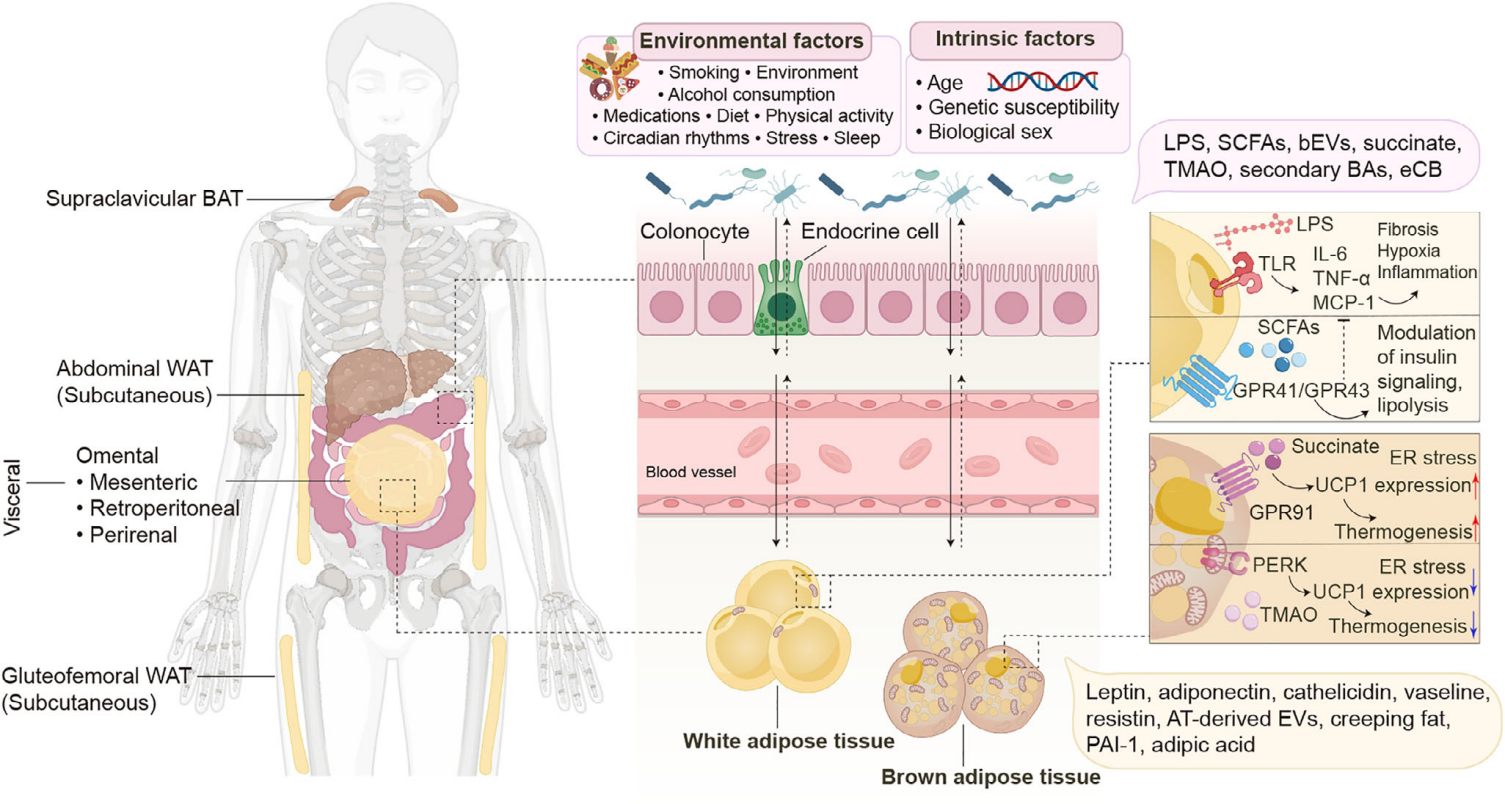
The molecular mechanisms of the gut–adipose tissue axis in the progression of metabolic syndrome. Human adipose tissue comprises distinct anatomical depots: subcutaneous WAT (abdominal and hip/thigh regions), visceral AT (surrounding abdominal organs), and thermogenic BAT and inducible beige adipocytes (primarily supraclavicular). Adipocytes secrete adipokines (leptin, adiponectin, adipsin, visfatin, vaspin, apelin, IL‐6, PAI‐1, FABP‐4) and ADEVs that modulate gut microbial composition. Reciprocally, gut microbiota‐derived factors regulate adipose tissue function: LPS activates TLR signaling to drive WAT inflammation, hypoxia, and fibrosis; SCFAs signal through GPR41/43 to modulate insulin sensitivity and lipolysis; and succinate signals through SUCNR1/GPR91, whereas TMAO‐related pathways may involve ER stress signaling, thereby influencing adipose remodeling and thermogenic regulation.

**FIGURE 4:**
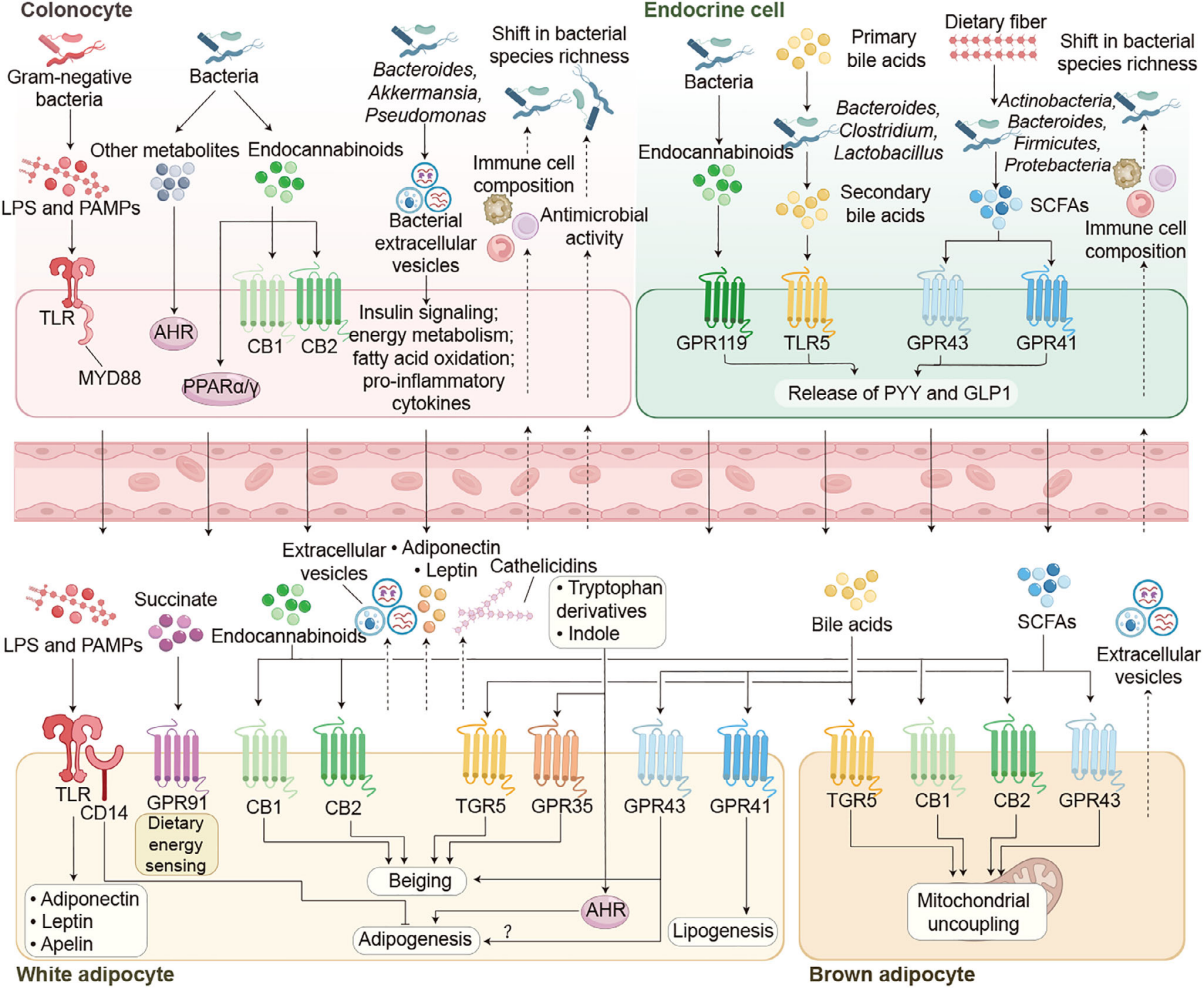
Molecular mechanisms in gut–adipose tissue crosstalk. Microbial components (LPS, PAMPs) and metabolites (SCFAs, bile acids, endocannabinoids, tryptophan derivatives) activate distinct receptor signaling in colonocytes (TLR‐MyD88, CB1/CB2, AhR, PPARα/γ) and enteroendocrine cells (GPR119, TGR5, GPR43/41), modulating intestinal barrier function and hormone secretion (GLP‐1, PYY, insulin). These molecules enter circulation to regulate white and brown adipocyte metabolism: TLR4/CD14 signaling controls adipokine production and adipogenesis; GPR91, AhR, and cannabinoid receptors govern energy sensing and adipogenesis; while TGR5 and GPR43/41 activation promotes WAT browning, mitochondrial uncoupling, and thermogenesis. Conversely, adipose‐derived factors (extracellular vesicles, adiponectin, leptin, cathelicidins) feedback to reshape gut microbial composition and immune homeostasis.

Microbially derived compounds, such as lipopolysaccharide (LPS), short‐chain fatty acids (SCFAs), bacterial extracellular vesicles (bEVs), succinate, trimethylamine N‐oxide (TMAO), secondary bile acids (BAs), and endocannabinoids (eCBs), can potentially be transported from the gut lumen into the bloodstream and ultimately into AT [28, 29]. The abnormal accumulation of these compounds may trigger inflammatory responses and contribute to metabolic syndrome (Figures 3 and 4). For example, transplantation of fecal microbiota from healthy lean donors into obese patients with Type 2 diabetes has been shown to increase butyrate‐producing bacteria, decrease total lipoprotein and low‐density lipoprotein (LDL) cholesterol (LDL‐C) levels, and reduce liver stiffness [30, 31]. Research has shown that germ‐free mice are less likely to gain weight on diet challenge and show improved glucose tolerance [32, 33]. Additionally, the gut microbiota can influence the development and function of AT in mice [34]. Specific microbial communities can promote or inhibit WAT browning or beige adipocyte recruitment, thereby enhancing energy expenditure in mice. For example, patients with obesity exhibit significantly lower fecal abundance of *Prevotella copri*. Administration of *Prevotella copri* can reduce fat deposition and promote browning in the inguinal WAT of mice with high‐fat diet (HFD)‐induced adiposity [35]. Therefore, regulating the gut microbiota through the use of prebiotics, probiotics, or synbiotics has emerged as a potential strategy for treating metabolic disorders. However, the metabolic effects of microbiota‐targeted interventions remain context dependent and require validation in well‐controlled metabolic disease cohorts. It is important to note that alterations in the gut microbiota of humans and mice are closely linked to environmental factors, including sleep, dietary intake, and physical activity [36]. More importantly, some prospective clinical studies suggest that the gut microbiota may be a major factor influencing energy metabolism by altering metabolites such as BAs, SCFAs, and bioactive lipids. For example, acetate supplementation, as an SCFA, increases the expression of genes involved in fatty acid biosynthesis and decreases protein levels related to lipid catabolism in WAT, thereby ameliorating streptozotocin‐induced hyperglycemia and AT loss [37].

Although many research findings emphasize the close association between specific gut microbiota taxa and health outcomes, many studies still remain at the level of correlation and fail to accurately establish causal relationships. Confounding of diet, medication use, or other lifestyle factors may affect microbial composition and clinical endpoints. Future research could use more targeted experiments, which may help clarify whether these microbial changes play a direct role in health regulation.

In summary, the gut microbiota is not only a key regulator of host metabolic health but also a sensitive hub that dynamically responds to changes in both the internal and external environments. Evidence from germ‐free mouse models and clinical intervention studies suggests that modulating the gut microbiota through diet, prebiotics, probiotics, or synbiotics may improve metabolic disorders in specific contexts. Future research should focus on elucidating how specific microbial communities and their metabolites precisely regulate AT function, thereby providing a theoretical foundation for personalized nutrition and precision medicine.

### 2.2 Systemic Consequences of Gut–AT Axis Dysregulation

#### 2.2.1 Insulin Resistance

Insulin resistance is the central metabolic defect in metabolic syndrome, characterized by impaired cellular responsiveness to insulin across multiple target tissues, including AT, skeletal muscle, liver, and endothelium [38]. While traditionally considered the primary initiating event, recent evidence suggests that insulin resistance is the convergent endpoint of multiple upstream pathological processes rather than a singular causative factor [39]. At the molecular level, insulin resistance involves disrupted phosphorylation cascades within the canonical insulin signaling pathway: serine phosphorylation of insulin receptor substrates (IRS)‐1 and ‐2—mediated by activated stress kinases such as c‐Jun N‐terminal kinase (JNK), inhibitor of nuclear factor kappa‐B (NF‐κB) kinase subunit beta (IKKβ), and mammalian target of rapamycin/S6 kinase (mTOR/S6K)—antagonizes normal tyrosine phosphorylation, thereby impairing phosphoinositide 3‐kinase (PI3K)–protein kinase B (Akt) activation and downstream metabolic responses [40, 41]. As a result, glucose uptake via glucose transporter Type 4 translocation is compromised in muscle and AT, while hepatic gluconeogenesis remains unsuppressed despite hyperinsulinemia. Lipid‐induced insulin resistance, or lipotoxicity, further exacerbates this dysfunction: elevated circulating free fatty acids, derived from dysfunctional AT, are preferentially esterified into diacylglycerols and ceramides in peripheral tissues, activating novel protein kinase C isoforms that phosphorylate and inactivate IRS proteins [42, 43]. Additionally, mitochondrial dysfunction, endoplasmic reticulum stress, and gut‐derived signals such as LPS collectively establish a self‐perpetuating cycle in which metabolic stress induces insulin resistance, which in turn amplifies metabolic inflexibility and promotes progression toward overt Type 2 diabetes mellitus [44].

#### 2.2.2 Chronic Low‐Grade Inflammation

Metabolic syndrome is increasingly recognized as a chronic inflammatory condition—termed “metaflammation”—characterized by persistent, low‐grade activation of innate and adaptive immune responses that both drive and perpetuate insulin resistance and metabolic dysfunction [45]. Unlike acute inflammatory responses, which are self‐limiting and protective, metaflammation is nutrient‐induced and fails to resolve, establishing a smoldering pathological environment. The nucleotide‐binding oligomerization domain‐like receptor protein 3 (NLRP3) inflammasome acts as a critical sensor of metabolic danger signals, including hyperglycemia, elevated saturated fatty acids, cholesterol crystals, and mitochondrial reactive oxygen species. Its assembly triggers Caspase‐1 activation and maturation of IL‐1β and IL‐18, potent proinflammatory cytokines that directly impair insulin signaling in metabolic tissues and promote pancreatic β‐cell apoptosis [46, 47]. Concurrently, the JNK and IKKβ/NF‐κB signaling pathways transduce inflammatory inputs from Toll‐like receptors (TLRs), cytokine receptors, and metabolic stressors [45].

AT, as both a source and target of inflammatory mediators, undergoes immune remodeling during metabolic syndrome. This macrophage phenotypic shift occurs predominantly within the stromal vascular fraction, especially in visceral fat depots, where immune cells, endothelial cells, preadipocytes, and stromal cells accumulate around hypertrophic or stressed adipocytes. Within this compartment, resident and recruited macrophages shift from anti‐inflammatory M2‐like states toward proinflammatory M1‐like states, accompanied by recruitment of CD8^+^ T cells, Th1 cells, and B cells that establish self‐sustaining inflammatory feedback loops through the production of TNF‐α, IL‐6, and MCP‐1 [48, 49, 50]. This chronic inflammatory state extends beyond metabolic tissues to promote endothelial dysfunction, accelerated atherogenesis, and increased thrombotic risk, thereby explaining the elevated cardiovascular and cerebrovascular morbidity associated with metabolic syndrome [51].

#### 2.2.3 Oxidative Stress

Oxidative stress is a fundamental mechanism underlying the pathogenesis of metabolic syndrome, occurring when the production of reactive oxygen and nitrogen species exceeds the capacity of cellular antioxidant defense systems [52]. In metabolic syndrome, excessive reactive oxygen species production originates from multiple sources: mitochondrial dysfunction characterized by impaired oxidative phosphorylation and electron leakage from respiratory chain complexes; activation of NADPH oxidase in response to inflammatory signals and hemodynamic stress; and uncoupled endothelial nitric oxide synthase, which generates superoxide instead of nitric oxide [53]. These reactive species damage lipids, proteins, and DNA, while also acting as signaling molecules that amplify pathological cascades—activating NF‐κB and AP‐1 transcription factors to drive inflammatory gene expression and further impairing insulin signaling through oxidative modifications of signaling intermediates [54]. The redox imbalance in metabolic syndrome is exacerbated by diminished antioxidant defenses, including reduced glutathione levels, superoxide dismutase activity, and catalase expression, creating a permissive environment for oxidative damage [55]. Importantly, oxidative stress establishes a bidirectional positive feedback loop with insulin resistance and inflammation: hyperglycemia and free fatty acids induce oxidative stress, which promotes insulin resistance and activates inflammatory pathways, which in turn generate additional reactive oxygen species [56]. This oxidative–inflammatory–insulin resistance triad propagates systemic metabolic dysfunction and contributes to complications such as endothelial dysfunction, progression of hepatic steatosis, and pancreatic β‐cell failure, highlighting redox modulation as a promising therapeutic target in managing metabolic syndrome.

#### 2.2.4 Epigenetic Modifications

Epigenetic modifications provide the mechanistic framework through which environmental exposures—including nutritional factors, physical activity, stress, and circadian rhythms—induce persistent alterations in gene expression without changing the DNA sequence. This process explains the developmental origins and transgenerational transmission of metabolic syndrome risk [57]. DNA methylation, the most extensively characterized epigenetic mark, exhibits global hypomethylation alongside site‐specific hypermethylation at promoters of genes regulating glucose and lipid metabolism, insulin signaling, and inflammatory responses in metabolic tissues of individuals with metabolic syndrome. These methylation patterns are established in utero in response to maternal nutrition and persist throughout life, predisposing offspring to metabolic disease [58]. For example, a meta‐analysis of the epigenome revealed relationships between adipokines and extensive, function‐specific gene regulation differences, which play a central role in Type 2 diabetes and its risk factors [59]. Histone modifications—including acetylation, methylation, phosphorylation, and ubiquitination—further regulate chromatin accessibility and transcriptional activity. Histone acetyltransferases and deacetylases (particularly sirtuins responsive to NAD^+^/NADH ratios) link the cellular metabolic state directly to epigenetic control [60]. Noncoding RNAs, including microRNAs, long noncoding RNAs, and circular RNAs, posttranscriptionally regulate entire gene networks implicated in metabolic syndrome. Specific microRNAs, such as miR‐103/107, which promote insulin resistance, and miR‐34a, which impairs adipose browning, serve as both pathogenic mediators and circulating biomarkers [61, 62]. The reversible nature of epigenetic modifications presents attractive therapeutic opportunities: histone deacetylase (HDAC) inhibitors, DNA methyltransferase modulators, and microRNA‐based therapeutics are under active investigation. Additionally, lifestyle interventions—including dietary modification and physical activity—exert beneficial effects partly through favorable epigenetic reprogramming [63, 64]. This environmental–epigenetic–metabolic axis underscores the potential for early‐life intervention and precision prevention strategies in combating the metabolic syndrome epidemic.

Overall, metabolic syndrome results from a complex interplay of insulin resistance, chronic metaflammation, oxidative stress, and epigenetic modifications. In this process, AT dysfunction, gut‐derived signals, and environmental exposures create self‐perpetuating pathological cycles that exacerbate metabolic dysfunction across multiple organs. These interconnected mechanisms—including mitochondrial dysfunction, inflammasome activation, redox imbalance, and heritable epigenetic reprogramming—collectively drive disease progression and offer multiple therapeutic targets for precision intervention. While these systemic mechanisms define the pathophysiological landscape, organ‐specific crosstalk—particularly the gut–AT axis—provides an integrative framework for understanding the progression of metabolic syndrome.

## 3 Adipose‐Derived Signals in Gut–AT axis

Recent research indicates that AT, including WAT, beige AT, and BAT, plays a crucial role in regulating the host's immune response and interacts with the gut microbiota. This interaction influences the intestinal environment and the ecological balance of the microbiota, thereby contributing to the progression of metabolic syndrome [65, 66]. Although the molecular mechanisms underlying these interactions are not yet fully understood, evidence supports the existence of this crosstalk. Notably, white and brown adipocytes release adipose‐derived factors, such as extracellular vesicles and adipokines, which may affect intestinal function (Figure 3). These signaling molecules serve as key mediators in the bidirectional communication along the gut–AT axis and are the focus of the following subsections (Figure 2).

### 3.1 Extracellular Vesicles and Adipokines in Gut–AT Axis

Communication between AT and the gut is significantly mediated by secreted factors, with extracellular vesicles and adipokines emerging as key communicators. Similar to how gut bacteria utilize bEVs for information exchange, both brown and white adipocytes release extracellular vesicles containing bioactive substances, such as microRNAs, which can influence protein expression in distant tissues, including the intestine [67]. In fact, while nearly all cell types in the human body can release extracellular vesicles, studies indicate that approximately 80% of circulating extracellular vesicles in the blood originate from adipocyte‐derived vesicles [67]. ADEVs are membranous nanoparticles that mediate communication from AT to other organs. IBD is an inflammation‐mediated condition that results in gut dysbiosis and inflammation of visceral AT. Visceral AT‐derived exosomes exacerbate colitis severity via proinflammatory microRNAs in HFD‐fed mice, confirming the existence of this exosomal pathway between AT and the intestinal *lamina propria* [68]. Additionally, exosomes secreted by adipose‐derived mesenchymal stem cells alleviate dextran sulfate sodium‐induced acute colitis by inducing regulatory T cells (Tregs) and reducing inflammatory cytokines (IFN‐γ, TNF‐α, IL‐12, and IL‐17) [69, 70, 71]. These findings demonstrate that AT‐derived extracellular vesicles can reshape the intestinal immune environment and barrier function, thereby reinforcing bidirectional communication between AT and the gut.

Adipokines are peptides that communicate the functional status of AT to targets in the gut. The secretion of adipokines, including leptin and adiponectin, is altered in cases of AT dysfunction and may contribute to metabolic disorders. Supplementation with leptin and adiponectin can regulate the relative abundance of specific bacterial species in the gut, likely by modulating the host's inflammatory response and enhancing the barrier function of the intestinal epithelium. These findings further support the concept of interactions between adipokines and gut microbiota [72]. Additionally, subcutaneous WAT may directly interact with bacteria by releasing cathelicidin, an antimicrobial peptide that exerts its effects by disrupting bacterial cell membranes [73]. Furthermore, other adipokines, such as PAI‐1 [74], adipsin, visfatin, vaspin, and resistin [75], may have specific effects on gut microbiota, either directly or indirectly, by influencing immune system regulation. Whether subcutaneous WAT can release substances with antibacterial effects remains an area requiring further investigation.

In summary, ADEVs and adipokines act as crucial mediators through which AT communicates with the gut, influencing inflammation, barrier integrity, and microbiota composition. This bidirectional flow of information underscores the complex nature of the gut–AT axis, where dysfunction in one organ can affect the other, contributing to metabolic disorders.

### 3.2 AT Contains Gut Bacteria

Beyond signaling molecules, the physical translocation of gut bacteria into AT depots, particularly visceral fat, represents a direct and intriguing aspect of the gut–adipose connection, especially evident in pathological conditions such as Crohn's disease. The discovery of “creeping fat,” or visceral AT surrounding the mesentery in patients with Crohn's disease, further confirms the interrelationship between AT and the microbiota [76]. The migration of intestinal bacteria to mesenteric AT occurs in both healthy individuals and Crohn's disease patients; however, notable differences exist in the bacterial populations present in visceral AT between these groups [77, 78]. Under normal conditions, *Clostridium innocuum—*a Gram‐positive, anaerobic, spore‐forming bacillus—has been identified as part of the normal intestinal microbiota [79]. In IBD, the intestinal mucosal barrier function is impaired, allowing substances from the intestine to infiltrate the bloodstream, which leads to an increased abundance of *Clostridium innocuum*. This bacterium serves as a signature member of this consortium, exhibiting strain variation between mucosal and adipose isolates, suggesting a preference for lipid‐rich environments. The proliferation and fibrotic processes associated with creeping fat attract macrophages and produce related inflammatory factors, thereby preventing the systemic spread of intestinal bacteria [80, 81]. This mechanism indicates that visceral AT is a complex, dynamic structure capable of responding to physiological processes such as increased intestinal permeability—a common feature of IBD and metabolic syndrome—and may exhibit specific responses to microbial signals.

The impact of gut microbiota present in creeping fat on other fat depots and disease states remains unclear. While some studies have suggested that AT in the human body contains no cultivable bacteria and that 16S ribosomal RNA has not been detected, specialized research on the AT of severely obese patients undergoing weight loss surgery has revealed the presence of cultivable bacterial communities and bacterial genes in multiple visceral AT depots [82, 83]. Furthermore, a recent comparative analysis of the microbial characteristics of AT in patients with Type 2 diabetes versus nondiabetic individuals identified specific traits associated with Type 2 diabetes in mesenteric fat, characterized by decreased bacterial diversity and an increase in certain gram‐negative bacteria. Although growing evidence suggests that bacterial DNA may not necessarily indicate the presence of live bacteria and that bacterial DNA could be transferred from the gut to fat storage depots, further research with stringent contamination controls is essential to validate these findings [84].

Together, these findings suggest that adipose‐associated microbial signals may represent a direct but still incompletely validated bridge between gut barrier dysfunction, local adipose inflammation, altered adipokine secretion, and systemic metabolic dysregulation.

## 4 Intestinal Signals Regulating AT Function

Recent research has demonstrated that certain microbial metabolites and other bioactive compounds significantly influence AT, complementing the previously established global correlations between entire microbial communities or specific bacteria and AT functionality (Figure 2) [85]. Gut‐derived signals can be functionally grouped into three interconnected modules: energy sensors and metabolic rheostats, inflammatory triggers and immune modulators, and bioactive lipid mediators and thermogenic modulators (Table 1). Energy sensors and metabolic rheostats mainly include fasting‐induced adipose factor (FIAF)/ANGPTL4, SCFAs, BAs, and succinate, which regulate lipid uptake, adipogenesis, thermogenesis, mitochondrial activity, and systemic energy balance. Inflammatory triggers and immune modulators, including LPS, pathogen‐associated molecular patterns (PAMPs), heat shock proteins (HSPs), and tryptophan‐derived metabolites, connect gut barrier dysfunction with innate immune activation, ER stress, and adipose metaflammation. Bioactive lipid mediators and thermogenic modulators, such as eCBs and oxylipins, further link gut microbiota, intestinal barrier integrity, adipose inflammation, and thermogenic remodeling. This functional classification highlights how intestinal signals regulate AT through coordinated metabolic, inflammatory, and lipid‐signaling pathways rather than acting as isolated metabolites (Figure 3).

**TABLE 1:** Microbial compounds in gut–AT axis in the progression of metabolic syndrome.

Compound	Mechanism	Source bacteria	Diseases	Target	Adipose effects	References
bEVs	Spreading and distributing throughout the body in intestinal epithelial cells; interacting with receptors within the host's body to transport bEV cargo into the cell interior or fully integrate into the host's cytoplasm	Bacteroidetes (*Bacteroides stercoris*); Akkermansia (*Akkermansia muciniphila*); and Pseudomonas (*Pseudomonas panacis*)	Obesity	Unknown	Decreasing adipogenesis through decreased the expression of C/EBPα and PPARγ; increasing energy metabolism and fatty acid oxidation through increased the expression of PPARα/γ; decreasing inflammation through decreased the expression of TNF‐α and IL‐6; increasing insulin resistance through increased the expression of pAkt	[86, 87, 88, 89]
		Bacteroidetes; Akkermansia; and Pseudomonas	MASLD		Relieving hepatic lipogenesis and fat accumulation	[89]
		*Akkermansia* and *Oscillibacter*	Diabetes		Decreasing TLR4 expression in AT; induce a stronger expression of ANGPTL4	[90]
		*Akkermansia muciniphila*; and *Escherichia coli*	Cardiovascular diseases		Inhibiting inflammatory responses and strengthening the intestinal barrier	[91]
SCFAs	Providing energy sources for intestinal epithelial cells, but also act as signaling molecules between host tissues and immune cells	*Actinobacteria*, *Bacteroidetes*, *Firmicutes*, *Proteobacteria*	Obesity	GPR41 and GPR43	Butyrate increases BAT thermogenesis and fat oxidation via vagal nerve signaling; decreases visceral AT inflammation and promote the release of leptin. Propionate inhibits adipocyte differentiation and fat accumulation. Acetate decreases BAT thermogenesis and increase WAT beiging.	[75, 92, 93, 94, 95]
			MASLD		Increasing in AT IGF‐1 production; activating thermogenic programs in BAT and WAT	[96, 97]
			Diabetes		Alleviating AT inflammation and inhibiting adipose insulin resistance via the PI3K/AKT signaling pathway	[98]
			Cardiovascular diseases		Maintaining intestinal integrity, anti‐inflammation, modulating glucolipid metabolism, blood pressure	[99, 100]
Succinate	Influence systemic circulation and elevating levels are associated with proinflammatory conditions	*Bacteroidetes, Akkermansia, Veillonellae, Prevotella*	Obesity	GPR91	Inhibiting of lipolysis in WAT; activating of BAT and beige thermogenesis and clearance of systemic succinate.	[101, 102]
			MASLD		Increasing beige or BAT mass by UCP1	[103]
			Diabetes		Increasing UCP1‐dependent thermogenesis by BAT	[104]
			Cardiovascular diseases		Inhibiting adipose lipolysis, immunoinflammatory responses, oxidative stress, and energy metabolism	[105]
Secondary BAs	Promoting the digestion and absorption of cholesterol, triglycerides, and fat‐soluble vitamins	*Bacteroidetes*, *Clostridium*, *Lactobacillus*, *Ruminococcus*	Obesity	TLR5	Increasing BAT thermogenesis via thyroxine; increasing active thyroid hormone and insulin sensitivity	[106, 107, 108]
			MASLD	FGF15	Increasing in thermogenesis in BAT	[109]
			Diabetes	TGR5	Activating the adipose TGR5 and upregulated expression of UCP1, resulting in elevation of WAT thermogenesis	[110]
			Cardiovascular diseases	TGR5	Decreasing body adiposity and serum lipids	[111]
TMAO	Bacteria containing choline compounds ferment to produce TMA, which is then converted into TMAO in the liver	*Clostridum, Eubacterium, Gammaproteobacterium*	Obesity	PERK	Increasing endoplasmic reticulum stress, inflammation, and decrease thermogenesis in BAT	[112]
			MASLD	Unknown	Causing AT inflammation; increasing proinflammatory cytokine MCP‐1 and decreasing anti‐inflammatory cytokine IL‐10 in AT	[113]
			Diabetes	Unknown	Increasing fat infiltration; downregulating the expression of perirenal AT	[114]
			Cardiovascular diseases	Unknown	Reducing BAT thermogenesis, lipogenesis and an improved specific fatty acid composition	[115]
eCBs	Regulating of energy metabolism, control of blood glucose and lipids, as well as roles in immune response, inflammatory response, and interactions between microbiota and host	*Bacteroidetes*, *Lactobacillus*, *Clostridium*	Obesity	CB1, CB2, GPR55, PPARγ	Increasing fat storage by inducing adipogenesis and triglyceride production; inhibiting lipolysis; increases expression of adipocyte differentiation genes	[116, 117, 118, 119]
			MASLD	CB1, CB2	Downregulating expression of its synthesizing enzymes Daglα and β in WAT and BAT; reducing liver fat	[120, 121, 122]
			Diabetes	CB2	Inhibiting VAT‐derived group 2 innate lymphoid cells; increasing inflammation in AT through their effector cytokines, and inhibiting ILC2 levels supports WAT	[122]
			Cardiovascular diseases	CB1, CB2	Decreasing BAT volume and glucose uptake	[123, 124]

Abbreviations: ANGPTL4, angiopoietin‐like 4; BAs, bile acids; BAT, brown adipose tissue; bEVs, bacterial extracellular vesicles; CB, cannabinoid receptor; C/EBPα, CCAAT/enhancer binding protein alpha; FGF15, fibroblast growth factor 15; GPR, G protein‐coupled receptor; pAKT, phosphorylated AKT; IGF‐1, insulin‐like growth factor 1; IL‐10, interleukin‐10; ILC2, group 2 innate lymphoid cell; MASLD, metabolic dysfunction‐associated steatotic liver disease; MCP‐1, monocyte chemoattractant protein‐1; PERK, protein kinase‐like ER kinase; PPAR, proliferator‐activated receptor; SCFAs, short‐chain fatty acids; TGR5, takeda G protein‐coupled receptor 5; TLR, Toll‐like receptor; TMA, trimethylamine; TMAO, trimethylamine N‐oxide; TNF‐α, tumor necrosis factor‐α; IL‐6, interleukin 6; UCP1, uncoupling protein 1;VAT, visceral adipose tissue; WAT, white adipose tissue.

Importantly, the strength of evidence supporting these gut‐derived signals varies substantially. Some pathways, such as LPS/TLR4‐mediated adipose inflammation and adipose‐specific N‐acylphosphatidylethanolamine‐hydrolyzing phospholipase D (NAPE‐PLD) deficiency in the eCB system, have relatively strong causal support from animal or genetic models. In contrast, many human findings on SCFAs, TMAO, BAs, and microbial community composition remain largely associative and may be influenced by diet, medication use, host genetics, disease stage, and adipose depot (Figure 4). Therefore, the following sections distinguish mechanistic evidence from animal or interventional models from correlative evidence in humans, while highlighting contradictory findings where they exist.

### 4.1 Energy Sensors and Metabolic Rheostats

#### 4.1.1 FIAF in Gut–AT Axis

FIAF/ANGPTL4 is a microbiota‐sensitive regulator of lipid metabolism in the gut–AT axis. FIAF, a member of the angiopoietin‐like protein family, is a circulating protein produced by various tissues—including the intestine, liver, and AT—under fasting conditions. This factor influences the metabolic response to fasting through activation of the peroxisome proliferator‐activated receptor (PPAR)α [125, 126, 127, 128].

FIAF plays a crucial role in regulating lipid metabolism in mice by inhibiting lipoprotein lipase (LPL), a key rate‐limiting enzyme responsible for hydrolyzing triglycerides in serum lipoproteins. By blocking LPL, FIAF reduces the absorption of fatty acids into AT and muscle [129, 130]. Furthermore, experiments conducted on mice have revealed that gut microbiota significantly influences FIAF production. In germ‐free mice, FIAF is expressed differently, and the normalization of the microbial community leads to a reduction in FIAF expression levels, an increase in LPL activity, and consequently, an increase in body fat. Additionally, germ‐free mice with *Angptl4* deletion exhibit heightened susceptibility to hyperlipidemia and atherosclerosis [131, 132, 133]. It is important to note that, although these findings are significant, caution is warranted when discussing the role of gut microbiota in the development of metabolic disorders. Research conducted on germ‐free mice over the past two decades has generated interest in the potential influence of gut microbiota on metabolic syndrome [134]. However, our understanding of the specific mechanisms involved remains limited. Current research challenges the prevailing belief that the absence of gut microbiota inherently confers immunity against metabolic syndrome. Variations in results may be attributed to the type of dietary fat consumed. Notably, a landmark study is unable to replicate the initial findings, leaving the question of whether the loss of gut microbiota affects metabolic syndrome unresolved. These findings underscore the need for further research and illustrate the complexity of the relationship between metabolic diseases and intestinal microbiota. There is ongoing debate regarding the role of FIAF synthesis in the fat storage effects associated with gut microbiota, particularly in germ‐free animals subjected to a HFD, where FIAF protein levels increase in the gut but not in the bloodstream [135].

According to several studies, administering specific bacteria to mice can increase their circulating levels of FIAF and enhance the expression of this protein in human intestinal epithelial cells [136, 137]. This finding suggests that the gut microbiota may regulate FIAF production. However, the precise mechanisms by which the gut microbiota modulate FIAF protein expression remain incompletely understood. Notably, FIAF appears to play a crucial role in the central hub of energy metabolism—the mouse hypothalamus—by inhibiting AMP‐activated protein kinase activity. It remains unclear whether the gut microbiota influence FIAF levels in the hypothalamus [138, 139, 140].

Overall, FIAF functions as a microbiota‐sensitive regulator of peripheral lipid uptake by inhibiting LPL, although its exact role in germ‐free models and potential involvement in the central nervous system require further investigation.

#### 4.1.2 SCFAs in Gut–AT Axis

SCFAs, primarily acetate, propionate, and butyrate, are major fermentation end‐products of dietary fiber produced by gut microbiota. These SCFAs serve as crucial multifunctional metabolites that link gut microbial activity with systemic metabolic regulation, including AT function. Because the human body lacks the enzymes necessary to break down dietary fiber, undigested carbohydrates reach the large intestine, where they are fermented by anaerobic bacteria, resulting in the production of SCFAs as primary metabolites. The quantity and type of dietary fiber consumed significantly influence the diversity and composition of the intestinal microbiota, thereby affecting SCFA production [141].

Studies suggest that abnormal increases or decreases in SCFA levels in humans and mice can adversely affect health, although the optimal concentration range remains unclear [142, 143]. The liver efficiently removes most circulating SCFAs to prevent elevated concentrations. In the liver, acetate serves as an energy source and precursor for cholesterol and long‐chain fatty acid synthesis, while propionate is utilized in gluconeogenesis. Low SCFA levels are associated with obesity, insulin resistance, diabetes, and other chronic metabolic diseases [144, 145]. Dietary fiber or SCFA supplementation can mitigate metabolic syndrome induced by HFD in rodent models through multiple physiological pathways [146, 147, 148]. Gastric bypass surgery increases circulating SCFAs, which is linked to increased BAT mass, activity, and overall energy expenditure. As signaling molecules, SCFAs—particularly butyrate and propionate—regulate the secretion of hormones critical for appetite, satiety, and energy expenditure. They stimulate the release of GLP‐1, peptide YY (PYY), and leptin [149, 150]. SCFAs bind to G protein‐coupled receptors GPR41 and GPR43 on intestinal endocrine L cells, promoting intestinal peptide production and initiating intracellular signaling pathways that influence energy metabolism, inflammatory responses, and insulin sensitivity in adipocytes, hepatocytes, neurons, and immune cells [151].

The relationship between SCFAs and AT function is complex and not yet fully understood, with research findings sometimes appearing contradictory. While some studies associate elevated SCFA concentrations with metabolic syndrome and insulin resistance, others report that SCFAs may improve insulin sensitivity and promote weight reduction in mice, rats, and humans [152, 153, 154]. For example, certain studies have found a positive correlation between increased SCFA concentrations and insulin resistance. Additionally, propionic acid serves as a substrate for gluconeogenesis in the liver, and excessive amounts may enhance hepatic glucose output, thereby exacerbating hyperglycemia [155]. Some cross‐sectional studies have observed that serum propionic acid levels in patients with Type 2 diabetes are higher than those in healthy controls, suggesting a potentially adverse role in specific pathological contexts [156]. In addition to receptor‐mediated signaling, butyrate can function as a HDAC inhibitor, thereby alleviating chronic low‐grade inflammation in AT and the liver and improving insulin signaling pathways [157]. Supplementation with butyric acid has been shown to significantly increase whole‐body insulin sensitivity in individuals with obesity and reduce visceral fat accumulation [158]. However, the mechanisms underlying butyrate's metabolic benefits are complex. Studies indicate that butyrate reduces food intake, prevents diet‐induced obesity, and activates BAT, increasing fatty acid oxidation and energy expenditure. These effects appear to be mediated through the gut‐brain neural circuit, as subphrenic vagotomy abolishes them [92].

Furthermore, different SCFAs exert distinct effects on AT metabolism through two partially distinct but complementary mechanisms: cell‐surface G protein‐coupled receptor signaling and intracellular epigenetic regulation [159]. First, SCFAs can regulate adipocyte differentiation and lipid handling through receptor‐mediated pathways involving GPR41 and GPR43. Butyrate promotes adipogenesis through activation of GPR43 [160, 161], whereas propionate stimulates adipogenesis in mature adipocytes via GPR41 activation [159, 162].

Binding of butyric acid to GPR43 upregulates adipogenic genes, including *Pparg* and *Cebpa*, which encode PPARγ and C/EBPα, thereby driving the differentiation of preadipocytes into mature adipocytes [163]. Propionic acid activates GPR41 to increase free fatty acid uptake, potentially involving the regulation of *Angptl4* expression, thereby enhancing LPL activity, promoting local deposition of circulating triglycerides, and stimulating lipid accumulation in mature adipocytes [159, 162]. GPR43 activation contributes to adipocyte differentiation and adipogenesis, whereas GPR41 may participate more broadly in adipocyte lipid handling and endocrine–metabolic responses [164].

The receptor‐mediated effects of SCFAs also appear to be species and depot dependent, particularly in thermogenic adipose depots. In vitro studies demonstrate that acetate promotes thermogenic and adipogenic gene expression, including *Fabp4/aP2*, *Ppargc1a*, and *Ucp1*, in mouse BAT, enhancing mitochondrial biogenesis in a GPR43‐dependent manner [165]. In contrast, in human WAT, GPR43 agonists have no effect on *aP2* gene expression or adipocyte differentiation in cultured preadipocytes. This suggests a potential species‐specific difference, as GPR43 in humans may not be directly linked to adipocyte differentiation as it is in mice [166]. Additionally, GPR43 expression in the AT of obese individuals correlates with inflammation, indicated by TNF‐α, rather than adipogenesis [167].

Second, SCFAs can also regulate AT biology through intracellular epigenetic mechanisms that are distinct from GPR41/43 signaling. Beyond receptor signaling, SCFAs also epigenetically regulate genes involved in metabolism and AT development through HDAC inhibition and modulation of DNA methyltransferases, influencing chromatin structure and DNA methylation [168]. These mechanisms contribute to maintaining metabolic homeostasis. Therefore, the adipogenic effects of butyrate through GPR43 activation and its epigenetic effects as an HDAC inhibitor should be interpreted as complementary but mechanistically distinct pathways.

Collectively, the inconsistent findings regarding SCFAs and AT function—ranging from beneficial metabolic improvements to associations with metabolic impairment—underscore the complexity of this interaction. From an evidence‐hierarchy perspective, current understanding is supported mainly by mechanistic studies in rodents and in vitro adipocyte models, whereas most human evidence remains associative. The effects of SCFAs appear to depend on the specific metabolite, receptor context, adipose depot, host species, dose, and metabolic state. Therefore, SCFA‐related findings should be interpreted as context dependent rather than uniformly beneficial or harmful. Identifying causal relationships and elucidating how individual SCFAs regulate adipogenesis, lipolysis, thermogenesis, and inflammation through receptor dependent or epigenetic mechanisms remain major challenges. Future studies combining tissue‐specific receptor knockout models, Mendelian randomization, longitudinal human cohorts, and detailed metabolite flux analyses will be essential to resolve these contradictions and clarify the role of SCFAs in the gut–AT axis.

#### 4.1.3 BAs in Gut–AT Axis

BAs, synthesized in the liver and modified by gut microbiota, serve as key signaling molecules that regulate AT metabolism, thermogenesis, and energy homeostasis through receptors such as the farnesoid X receptor (FXR) and Takeda G protein‐coupled receptor 5 (TGR5, also known as GPBAR1) [169]. Primary BAs—cholic acid (CA) and chenodeoxycholic acid (CDCA) in humans—are conjugated with taurine or glycine before secretion into the intestine, where they facilitate lipid absorption. Approximately 95% of BAs undergo enterohepatic recirculation, while the remainder are transformed by colonic bacteria into secondary BAs, such as lithocholic acid (LCA) and deoxycholic acid [170].

These microbially modified BAs function as metabolic regulators. Reduced levels of secondary BAs, such as LCA and ursodeoxycholate, in obesity‐prone mice are associated with downregulated GLP‐1 expression in the ileum and decreased expression of thermogenic genes, including *Ppargc1a* and *Ucp1*, in BAT, indicating suppressed thermogenic capacity [171]. In obese pig models, *Bifidobacterium pseudocatenulatum* mitigates fat accumulation by enhancing LCA biosynthesis [172]. Following bariatric surgery, increased taurine‐conjugated BAs improve systemic glucose regulation and reactivate BAT through intestinal FXR‐TGR5 signaling crosstalk [173].

Takeda G protein‐coupled receptor 5 (TGR5) is highly expressed in BAT and mediates metabolic effects, including mitochondrial biogenesis, β‐oxidation, and energy expenditure. Activation by BA analogs, such as INT‐777, enhances these processes in murine adipocytes [174, 175]. In humans, oral CDCA supplementation increases BAT activity and total energy expenditure in healthy females, with TGR5 agonists specifically enhancing mitochondrial coupling in primary human brown—but not white—adipocytes [176]. Furthermore, intestinal TGR5 signaling stimulates the release of PYY and GLP‐1 from L cells, linking BA signaling to appetite regulation and metabolic homeostasis [177].

In summary, gut microbiota‐derived secondary BAs modulate AT function through TGR5‐ and FXR‐dependent pathways, influencing thermogenesis, mitochondrial activity, and incretin secretion. These findings highlight the BA–gut–adipose axis as a promising therapeutic target for metabolic diseases.

#### 4.1.4 Succinic Acid in Gut–AT Axis

Succinic acid is a crucial component of the citric acid cycle, commonly known as the Krebs cycle or the tricarboxylic acid cycle, and plays a vital role in energy balance and cellular metabolism [178]. Various tissues, including the kidney, liver, heart, and retina, express succinate receptor 1 (SUCNR1/GPR91), a G protein‐coupled receptor to which succinic acid binds, influencing numerous physiological and pathological processes. Succinic acid interacts with GPR91 on adipose cells, helping regulate cellular metabolism [179]. Notably, succinic acid can be derived from carbohydrates through microbial fermentation and serves as a metabolic end product [180]. In microbial fermentation, propionate can be produced through the succinate pathway, which represents an important biochemical route for propionate generation. Additionally, succinic acid and propionic acid can be generated from the fermentation of amino acids [181]. The significance of succinic acid as a key microbial metabolite in promoting the beneficial metabolism of dietary fiber has been validated in mouse models: consumption of dietary fiber increases succinic acid levels produced by *Prevotella* [182]. Interestingly, the abundance of *Akkermansia muciniphila*, a bacterium that generates succinic acid as one of its main metabolites during mucin degradation, is negatively associated with IBD and obesity [183, 184, 185]. Furthermore, other succinic acid‐producing bacteria, such as *Parabacteroides distasonis*, have been shown to effectively ameliorate metabolic syndrome associated with abnormal metabolism in mice [186].

In mouse models of Type 2 diabetes, metabolic syndrome, and hypertension, elevated levels of succinic acid have been observed in the circulatory system compared with healthy controls [187]. However, unlike in mice and rats, these diseases in humans do not correlate with increased blood succinate concentrations [104]. Research indicates that the increased expression of the GPR91 receptor in WAT is associated with the regulation of fat mass and blood glucose levels. Studies using *Gpr91* gene knockout mice have shown that the absence of succinate receptors affects metabolism and body weight, although the specific effects vary depending on experimental conditions. Under standard dietary conditions, *Gpr91* knockout mice exhibit reduced adipocyte numbers, diminished WAT mass, increased energy expenditure, and improved glucose tolerance. Notably, the absence of *Gpr91* does not affect lipogenesis but leads to decreased adipocyte capacity and fat accumulation [188]. A more detailed examination of the metabolic changes resulting from *Gpr91* deficiency is conducted using maximal oxygen uptake testing. The results indicate that, compared with wild‐type mice, *Gpr91* knockout mice exhibit a lower oxygen consumption rate. When placed on an HFD, *Gpr91* knockout mice initially demonstrate resistance to obesity during the first few weeks. However, over time, these mice show increased fat accumulation, elevated blood glucose levels, reduced insulin secretion, and aggravated liver cell damage after 16 weeks compared with their wild‐type littermates [189]. These findings suggest that GPR91 may function as a dietary energy sensor and could represent a promising target for the treatment of diabetes, hypertension, and obesity.

Although there is currently no clear evidence directly linking Crohn's disease with overweight or obesity, findings indicate complex interactions among Crohn's disease, gut microbiota, and AT. Research has observed the accumulation of beige AT in patients with Crohn's disease [190]. Furthermore, this study shows that the plasma concentration of succinic acid in Crohn's disease patients is significantly higher than in healthy individuals. Compared with healthy individuals and patients with inactive Crohn's disease, those with active Crohn's disease exhibit elevated expression levels of GPR91 in WAT, adipose‐derived stem cells, and AT macrophages. Notably, in both the control group and patients with inactive Crohn's disease, succinate‐treated adipose‐derived stem cells show increased expression of several beige AT markers, including UCP1 [190]. Overall, these studies suggest that gut bacteria and succinate may promote beige remodeling of WAT or beige adipocyte recruitment in the context of Crohn's disease. Beige adipocytes are inducible thermogenic cells that emerge within WAT depots and exhibit metabolic properties distinct from both classical white adipocytes and constitutive BAT. Therefore, gut‐derived signals that promote beige remodeling may have depot‐specific effects, particularly in WAT regions exposed to inflammation, altered microbial metabolites, or local immune activation. However, further investigation is necessary to fully elucidate this transition process within the framework of metabolic syndrome.

Collectively, succinate—derived from both host metabolism and gut microbial fermentation (e.g., by *Akkermansia muciniphila* and *Parabacteroides distasonis*)—signals through GPR91 to exert complex and often paradoxical effects on AT and metabolism. While *Gpr91* deficiency in mice suggests its role as an energy sensor, the outcomes vary depending on diet and duration. Notably, elevated succinate levels and increased GPR91 expression in AT are associated with beige adipocyte accumulation in patients with Crohn's disease, indicating a potential context‐specific role in AT remodeling that differs from classic metabolic syndrome. These findings underscore the need for further research to elucidate the tissue‐specific and disease‐contextual roles of succinate signaling within the gut–AT axis.

### 4.2 Inflammatory Triggers and Immune Modulators

#### 4.2.1 LPS and PAMPs in Gut–AT Axis

LPS and other PAMPs are key mediators linking gut barrier dysfunction, microbial dysbiosis, and AT inflammation in metabolic syndrome. Metabolic endotoxemia—characterized by elevated circulating LPS—is associated with chronic low‐grade inflammation and insulin resistance [191, 192]. While an intact intestinal barrier normally prevents LPS translocation, factors such as a HFD, obesity, and low dietary fiber intake compromise barrier integrity, enabling LPS to enter systemic circulation [193, 194].

LPS activates innate immune signaling via TLR4 and its coreceptor cluster of differentiation 14 (CD14) in AT [195], triggering proinflammatory pathways involving TNF‐α and disrupting adipogenesis through suppression of PPARγ and CEBPα [196, 197]. This inflammatory state stabilizes β‐catenin via WNT–β‐catenin–T cell factor 4 (TCF4) signaling, further inhibiting preadipocyte differentiation [198, 199]. Additionally, LPS alters adipokine secretion by increasing leptin and apelin levels and promotes M1 macrophage polarization, contributing to AT dysfunction [200, 201]. Germ‐free mice are protected from high‐fat‐diet‐induced metabolic disturbances, underscoring the central role of gut microbiota in driving AT inflammation. However, the precise microbial components responsible remain unclear [133, 202, 203].

Notably, the effects of LPS are not uniform and depend on the structural and microbial context. For example, colonization with immunogenic *Escherichia coli* induces glucose intolerance and macrophage accumulation in WAT, whereas a low‐immunogenicity LPS‐producing strain (*E. coli* MLK1067) does not [204]. Similarly, LPS from Streptococcus pyogenes lacks the detrimental metabolic effects observed with E. coli LPS despite equivalent endotoxin units, highlighting the importance of lipid A acylation and molecular specificity beyond total LPS load [205].

Other PAMPs—including peptidoglycan recognized by nucleotide‐binding oligomerization domain protein 1 (NOD1) and bacterial lipoproteins detected via TLR2—also contribute to AT inflammation and lipolysis [206]. NOD1 stimulates endoplasmic reticulum stress kinases (ERK1 and ERK2) and protein kinase A pathways, which influence hormone‐sensitive lipase activity and promote lipid breakdown [206]. However, the role of these molecules in the development of metabolic diseases remains controversial. For instance, mice deficient in TLR2, TLR5, or NOD2 exhibit worsened metabolic profiles under HFD conditions, including increased adiposity, inflammation, and microbial translocation [207, 208, 209].

In summary, LPS and other PAMPs promote AT inflammation and metabolic dysfunction primarily through pattern recognition receptors such as TLR4 and NOD1/2. Their effects are highly context dependent, influenced by the bacterial source, molecular structure, and host factors. These findings underscore the importance of moving beyond measuring total LPS levels to focus instead on specific microbial signatures and host–pathogen interactions in metabolic diseases.

#### 4.2.2 HSPs in Gut–AT Axis

HSPs, specifically HSP70 and glucose‐regulated protein‐78 (GRP78), are released from the jejunal mucosa and function as proinflammatory signals [210]. Several studies have shown that circulating levels of GRP78 are elevated in individuals with insulin resistance, hyperglycemia, and MASLD. Furthermore, mice with a partial knockout of GRP78 are protected against diet‐induced obesity. GRP78 plays a role in adipogenesis, lipid droplet stabilization, insulin resistance, and liver steatosis. The regulation of GRP78 following metabolic surgery highlights the bypass of the small intestine as a critical factor influencing GRP78 secretion [211, 212].

Overall, HSPs such as GRP78 connect intestinal stress and inflammation to AT dysfunction and metabolic disease, underscoring an additional pathway through which gut‐derived signals influence adipose biology.

#### 4.2.3 Tryptophan Derivatives in Gut–AT Axis

Tryptophan metabolism, orchestrated by both gut microbiota and host cells, generates a spectrum of bioactive derivatives such as indoles, kynurenine (Kyn), and kynurenic acid (Kyna), which serve as critical signaling molecules regulating AT function and systemic metabolism. Tryptophan can be converted into various metabolic molecules by intestinal microbiota and adipocytes. For instance, indole, a tryptophan metabolite produced by bacteria, includes indole‐3‐propionic acid, which is typically found at lower concentrations in the bloodstream of obese individuals compared with those of normal weight [213]. Tryptophan degradation primarily occurs via the Kyn pathway, leading to the production of Kyn, Kyna, and quinolinic acid. Conversely, evidence indicates that obese individuals have elevated plasma levels of Kyn, potentially associated with increased activity of indoleamine 2,3‐dioxygenase 1 (IDO1) [214]. Additionally, some enzymes encoded by gut bacteria share homology with enzymes in the Kyn pathway found in eukaryotes. Tryptophan derivatives, including indole metabolites, can activate aryl hydrocarbon receptor (AhR) signaling, thereby influencing adipocyte differentiation and AT remodeling [215, 216]. The metabolic processes of adipocytes and fat accumulation are modulated by the AhR signaling system. However, the primary source of Kyn and its impact on metabolic syndrome have not been thoroughly investigated. Other studies indicate that Kyna promotes fatty acid oxidation, thermogenesis, and the transcription of anti‐inflammatory factors in AT by activating GPR35 receptors, which inhibits weight gain in mice fed a HFD and improves their glucose tolerance.

Kyna–GPR35 signaling enhances *Ppargc1a* and cellular respiration‐related gene expression in adipocytes and increases *Rgs14* expression, thereby amplifying β‐adrenergic receptor signaling [217]. In contrast, mice with *Gpr35* deletion exhibit weight gain, decreased glucose tolerance, and increased susceptibility to HFD. Additionally, these mice show impaired exercise‐induced browning of AT [218]. These findings highlight a novel pathway through which metabolic products of the gut microbiota communicate with the host to regulate energy balance. As previously mentioned, the activity of the IDO1 enzyme increases in metabolic syndrome. However, its specific role in metabolic diseases requires further investigation. Studies in both mice and humans have demonstrated that metabolic syndrome is associated with heightened IDO1 activity in the gut, which shifts tryptophan metabolism from the production of indole derivatives and IL‐2 toward Kyn production [219]. Moreover, research has shown that genetic deletion of *Ido1* or pharmacological inhibition of IDO1 can enhance insulin sensitivity, maintain intestinal mucosal health, reduce metabolic endotoxemia and inflammation, and regulate lipid metabolism in the liver and AT [220].

Research data indicate that, in addition to intestinal bacteria, AT may serve as a primary direct source of Kyn. Adipocytes express the *Ido1* gene and its corresponding IDO1 protein, as demonstrated in vivo [221]. Inhibiting IDO1 in adipocytes can protect mice from metabolic syndrome by preventing the accumulation of Kyn. It is important to note that the physiological mechanism underlying this effect also involves activation of the AhR, as the impact of Kyn is diminished when AhR is absent from adipocytes [221]. Furthermore, the study reveals that the tryptophan metabolites produced by gut microbiota can regulate the expression of the miR‐181 family in mouse white adipocytes, which subsequently affects insulin sensitivity and energy expenditure. In mice, metabolic syndrome, insulin resistance, and inflammatory processes in WAT are also associated with the dysregulation of the miR‐181 axis within the intestinal microbiome. This is further supported by research conducted on a pediatric population categorized by weight percentile, which finds that obese individuals exhibit imbalanced serum concentrations of tryptophan metabolites and altered miR‐181 expression in WAT [222].

Collectively, tryptophan metabolites—including indoles, Kyn, and Kyna—act through distinct receptors such as the AhR and GPR35 to influence adipocyte differentiation, thermogenesis, inflammation, and insulin sensitivity. Dysregulation of this pathway, characterized by a shift toward increased Kyn production driven by upregulated IDO1 in both the gut and AT and away from beneficial indoles, is a hallmark of metabolic dysfunction. Targeting specific branches of tryptophan metabolism or their receptors—such as IDO1 inhibition or GPR35 activation—holds therapeutic potential but requires a deeper understanding of AT–specific effects and the complex interplay between microbial and host‐derived metabolites.

### 4.3 Bioactive Lipid Mediators and Thermogenic Modulators

Bioactive lipids comprise a diverse group of signaling molecules derived from fatty acids, phospholipids, and sphingolipids, playing pivotal roles in metabolic regulation, inflammation, and the bidirectional communication between gut microbiota and AT [223]. These lipids are involved in various biological functions, including the modulation of inflammatory responses, pain perception, blood pressure regulation, cell development and differentiation, apoptosis, and immune responses. The host's metabolic processes can be influenced by the composition and activity of the microbial community, which, in turn, is affected by bioactive lipids produced within the body and metabolites generated by the gut microbiota [224].

#### 4.3.1 eCBs in Gut–AT Axis

The eCB system, comprising endogenous ligands such as anandamide and 2‐arachidonoylglycerol (2‐AG) and receptors cannabinoid Type 1 (CB1) and Type 2 (CB2), forms a critical bidirectional link between gut microbiota composition, intestinal barrier integrity, and AT metabolism in obesity and metabolic syndrome. The eCB system serves numerous physiological functions, including energy regulation, control of circulating glucose and lipid levels, as well as roles in immune and inflammatory responses and interactions between the microbiota and the host [225]. In 1988, the first endogenous cannabinoid receptor, CB1, was identified and found to be activated by Δ9‐tetrahydrocannabinol, the psychoactive component of cannabis [226]. In 1993, the second receptor, CB2, was also confirmed [227]. Both receptors utilize similar signaling pathways and belong to the G protein‐coupled receptor family. Anandamide, the first endogenous cannabinoid identified, binds to both CB1 and CB2 receptors and is classified as a member of the N‐acylethanolamine bioactive lipid family [228]. The next endogenous cannabinoid receptor ligand identified was 2‐AG [229]. Following the discovery of these two primary molecules, additional compounds with specific affinities for CB1 and CB2 receptors have been incorporated into the eCB family. Numerous recent studies in humans and rodents indicate that the eCB system plays a significant role in the metabolic processes of AT and that its activation promotes fat accumulation [230, 231].

Subsequent research has demonstrated that the eCB system plays a critical role in maintaining the integrity of the intestinal barrier, influencing gut microbiota composition, and regulating AT metabolism [232]. Specifically, a mouse study observed that under conditions of metabolic syndrome and diabetes, anandamide levels increased, which in turn heightened intestinal permeability via a CB1 receptor (CB1R)‐dependent pathway. Furthermore, activation of the eCB system induced by medication may lead to increased fat synthesis and disruption of the intestinal barrier. The integrity of the intestinal barrier may be compromised, and the stability of the eCB system in both the intestine and AT may be affected, as the rise in intestinal permeability also elevates blood levels of LPS. In the pathological state of metabolic syndrome, imbalance of the eCB system and increased LPS levels contribute to abnormalities in lipogenesis, perpetuating the initial imbalance and creating a vicious cycle that ultimately results in alterations in AT metabolism [233]. This discovery elucidates the connection between intestinal microbiota and the eCB system, emphasizing the crucial role of the eCB system in regulating adipose lipid accumulation. Moreover, it indicates that adipose lipid accumulation is modulated by a feedback loop involving LPS and the eCB system. These changes may contribute to dysfunction of the eCB system or the development of a vicious cycle in obesity, as metabolic syndrome is often associated with alterations in the eCB system. Such alterations may include changes in eCB levels, expression of CB1 and CB2 receptors, enzyme activity related to eCB synthesis and degradation, increased blood LPS levels, disruption of intestinal microbiota composition, and impairment of AT metabolic processes [200].

Based on these findings, subsequent studies involving additional mice have confirmed the connection between the eCB system, gut microbiota, and AT metabolism. The researchers observed significant alterations in the gut microbiota associated with hereditary metabolic syndrome and diabetes, conditions closely linked to changes in overall tissue metabolism and eCB system function [234]. Similar results have also been observed in diet‐induced obesity and germ‐free mice. Overall, these findings provide compelling evidence of the reciprocal interactions among unique bioactive lipids, gut microbiota, AT metabolism, and the intestinal functions of the eCB system [235].

Ultimately, investigators have developed several mouse models specifically lacking NAPE‐PLD, a crucial enzyme that regulates the production of bioactive lipids in AT. This research aimed to clarify the relationship between adipose bioactive lipid production, metabolic disorders, and gut microbiota alterations. Even when maintained on a standard‐calorie diet, mice deficient in NAPE‐PLD, particularly in their AT, exhibit spontaneous weight gain, insulin resistance, and inflammatory conditions. Furthermore, these mice are more susceptible to metabolic diseases induced by an HFD. The thermogenic process in AT, often referred to as browning or beiging, is diminished when NAPE‐PLD is specifically deleted from adipocytes. This alteration also significantly impacts the composition of the gut microbiota. The entire phenotype, including reduced browning or beiging, is replicated when the intestinal microbiota from NAPE‐PLD‐deficient mice is transplanted into recipient mice, suggesting that the gut microbiota plays a causative role [236].

Overall, these findings consistently indicate a bidirectional relationship between the eCB system and gut microbiota. However, to fully understand this relationship, further in‐depth investigation is necessary. A recent study suggests that gut microbiota may produce specific N‐acyl amides structurally similar to human GPCR ligands, as demonstrated by bioinformatics analyses of individual microbiota [116]. In an oral glucose tolerance test, germ‐free mice colonized with bacteria expressing N‐acyl serinol synthase exhibit lower blood glucose levels, consistent with the effect of these bacteria on the host GPR119 receptor. This discovery opens new avenues for studying microbiota‐host interactions and offers potential opportunities for developing therapeutic targets [116]. These findings provide mechanistic support for microbiota‐host lipid signaling as a potential therapeutic target. However, direct causal evidence in humans remains limited, and most clinical data support associations among eCB tone, gut barrier dysfunction, adiposity, and metabolic risk. Thus, the gut–adipose eCB axis should be regarded as a mechanistically supported but still clinically unproven mediator of metabolic syndrome.

#### 4.3.2 Oxylipins in Gut–AT Axis

Oxylipins are bioactive lipid mediators generated from polyunsaturated fatty acids and participate in inflammatory and metabolic signaling relevant to metabolic syndrome [237]. 12,13‐Dihydroxyoctadecadienoic acid (12,13‐diHOME, also referred to as isoleukotoxin diol), is produced through the metabolism of linoleic acid by cytochrome P450 and soluble epoxide hydrolase [238]. It is predominantly synthesized in BAT or beige AT, and its concentration is regulated by factors such as exercise, nutrition, and ambient temperature. 12,13‐diHOME influences fatty acid uptake in AT and helps regulate body temperature in response to cold exposure. In a cohort of 28 obese adolescent males, 12,13‐diHOME levels are found to be lower than those in 28 age‐matched normal‐weight males, with levels significantly increasing following acute exercise [239]. In a mouse model of obesity induced by a HFD, continuous administration of 12,13‐diHOME for 2 weeks promotes fatty acid transport to BAT, reduces circulating triglyceride levels, and upregulates the expression of the LPL gene in BAT. Notably, certain gut bacteria have been identified as capable of synthesizing and secreting 12,13‐diHOME [240]. For instance, *Dysosmobacter welbionis* can produce this bioactive lipid, and its metabolites have been recognized as 12,13‐diHOME. In mouse studies, treatment with this bacterium significantly mitigates HFD‐induced whitening of BAT and enhances mitochondrial activity [241, 242].

Overall, oxylipins such as 12,13‐diHOME, produced by both brown/beige AT and specific gut bacteria (e.g., *Dysosmobacter welbionis*), play a functional role in regulating fatty acid uptake by AT and promoting thermogenesis. The association of lower 12,13‐diHOME levels with obesity, along with their increase following exercise, suggests their involvement in metabolic health. This highlights the gut microbiota as a potential source of beneficial oxylipins that target AT metabolism.

### 4.4 Other and Emerging Gut‐Derived Modulators and Translational Considerations

In addition to the major intestinal signals discussed above, recent attention has also been drawn to emerging gut‐derived modulators such as imidazole propionate, urolithin A, and TMAO which connect microbial metabolism with adipose inflammation, insulin sensitivity, thermogenesis, or systemic cardiometabolic risk. Although many of these molecules have not yet been fully integrated into the gut–AT axis framework, they may represent important candidates for future mechanistic and therapeutic studies.

Imidazole propionate, a gut microbiota‐derived histidine metabolite, has emerged as a potential mediator of insulin resistance. Elevated imidazole propionate levels have been reported in individuals with Type 2 diabetes, and mechanistic studies show that imidazole propionate impairs insulin signaling by activating the p38γ–p62–mTORC1 pathway at the level of IRS. Although most evidence currently links imidazole propionate to hepatic and systemic insulin resistance rather than direct adipocyte‐specific effects, this metabolite may indirectly aggravate AT dysfunction by promoting whole‐body insulin resistance and low‐grade metabolic inflammation [243].

Urolithin A, a gut microbiota‐derived metabolite produced from dietary ellagitannins, has been proposed as an emerging thermogenic and mitochondrial regulator. Experimental studies in mice indicate that urolithin A increases energy expenditure by enhancing BAT thermogenesis and promoting WAT browning. These effects have been linked to mitochondrial biogenesis, improved metabolic flexibility, and activation of thermogenic programs. Therefore, urolithin A may represent a microbiota‐dependent postbiotic candidate that connects diet, gut microbial transformation, and adipose energy expenditure [244].

TMAO should be interpreted as a risk‐associated host–microbial cometabolite rather than a uniformly causal adipose regulator. Human studies mainly report associations between circulating TMAO levels and obesity, insulin resistance, abdominal adiposity, vascular dysfunction, or cardiovascular risk, whereas direct evidence that TMAO causally remodels AT remains limited and context dependent [245]. Therefore, its role in the gut–AT axis should be discussed cautiously, particularly when extrapolating from systemic cardiometabolic associations to adipose‐specific mechanisms.

Together, these emerging modulators highlight a broader translational issue: adipose responses to gut‐derived signals are highly species and depot dependent. Findings from mouse models, particularly those involving BAT activation, WAT browning, beige adipocyte recruitment, or GPR41/43 signaling, should not be directly extrapolated to humans without validation. For example, SCFA–GPR43 signaling may promote adipogenic and thermogenic programs in mouse adipocytes, whereas human WAT studies suggest weaker adipogenic effects and closer associations with inflammation. Similarly, visceral AT, subcutaneous AT, BAT, and beige adipocytes differ in immune composition, thermogenic capacity, lipid‐buffering ability, and responsiveness to gut‐derived metabolites. These differences are particularly relevant for beige adipocytes, which emerge within WAT depots and may respond selectively to signals such as SCFAs, BAs, succinate, and urolithin A. Therefore, species, adipose depot, disease stage, and cellular composition should be considered when interpreting gut–AT axis studies and translating them into therapeutic strategies.

## 5 Therapeutic Strategies for Metabolic Syndrome

In managing metabolic syndrome, lifestyle modifications should be integrated with targeted pharmacotherapy to optimize cardiovascular risk reduction. Accumulating evidence underscores the clinical benefits of combining lifestyle interventions with established drug therapies—or using pharmacologic agents alone—to effectively control risk factors and reduce cardiovascular events in patients with Type 2 diabetes, hypertension, dyslipidemia, and chronic kidney disease. Moreover, emerging therapeutic strategies directed at the gut–AT axis have demonstrated promising efficacy. These gut–AT axis interventions primarily encompass lifestyle modifications and microbiome‐targeted approaches, including probiotics, FMT, surgical procedures, and pharmacologic agents targeting specific signaling pathways or microbial metabolites (Table 2).

**TABLE 2:** Clinical trials for metabolic syndrome.

Therapeutic approaches	Disease	Effects	Registration number	References
Diet				
Mediterranean diet	MASLD	Decreasing fat mass, visceral AT, subcutaneous abdominal AT, insulin, glycated hemoglobin, and blood pressure	ChiCTR2200064547	[111]
Mediterranean diet and pentadecanoic acid	Fatty liver disease	Lowering LDL‐C and increasing the abundance of Bifidobacterium adolescentis	NCT05259475	[246]
Low‐energy diets	Overweight and prediabetes	Influencing gut microbiota and individual taxa and total body fat and body weight	NCT01777893	[247]
The multicomponent weight loss maintenance 3 phases program	Obesity	Increasing levels of Faecalibacterium and reducing in fat mass and visceral adiposity	NCT04192357	[248]
Interrupting prolonged sitting with intermittent walking	Overweight and obesity	Not significant changing AT dipeptidyl peptidase 4 secretion	NA	[249]
Energy restriction and vigorous‐intensity exercise	Overweight and obesity	Significantly reduced body mass, fat mass, fasted insulin, leptin, and total cholesterol	NA	[250]
Time‐restricted eating and prebiotic	Pediatric cancer survivors	Decreasing in fat mass and visceral fat mass	NCT05826184	[251]
Intermittent calorie restriction	Overweight and obesity	Remaining the gut microbiome stable and highly individual specific	NCT02449148	[252]
Time‐restricted eating	Healthy volunteers without obesity	Improving fasting glucose, reducing total body mass and adiposity, ameliorating inflammation, and increasing gut microbial diversity	ChiCTR2000029797	[253]
Prebiotics				
Oligofructose‐enriched inulin	MASLD	Decreasing in percent trunk fat	NCT02568605	[254]
Camu‐camu	Overweight and hypertriglyceridemia	Decreasing liver fat	NCT04130321	[255]
Human milk oligosaccharide 2′‐fucosyllactose	Overweight	Reducing the body fat and less loss of the fat‐free mass	NCT06547801	[256]
Metformin	Obesity and diabetes	Influencing the enrichment of *Dysosmobacter welbionis*, reducing body weight gain and liver weight, and improving glucose tolerance	NCT03852069	[257]
Dibifree	Type 2 diabetes	Improving glycemic control and reducing adiposity	NCT06224803	[258]
Probiotics				
*Lactobacillus plantarum* LMT1‐48	Obesity	Decreasing body fat mass	KCT0008977	[259, 260]
*Lactobacillus plantarum* K50	Obesity	Reducing visceral adiposity	KCT0003944	[261]
*Lactiplantibacillus plantarum* SKO‐001	Obesity	Decreasing the subcutaneous fat area, total cholesterol levels, and leptin levels	KCT0008871	[262]
*Lacticaseibacillus paracasei* BEPC22 and *Lactiplantibacillus plantarum* BELP53	Overweight	Reducing body fat and weight	KCT0007268	[263]
*Lactobacillus acidophilus*, *Lactobacillus plantarum*, *Bifidobacterium lactis*, and *Saccharomyces boulardii*	Type 2 diabetes	Decreasing in adiposity (waist circumference)	NA	[264]
*Lactobacillus acidophilus*, *Bifidobacterium lactis*, *Bifidobacterium longum*, *Lactobacillus casei*, *Lactobacillus rhamnosus*, *Lactobacillus plantarum*, *Lactobacillus salivarius*, *Bifidobacterium brevis*, and microcrystalline cellulose excipient	hypertension and overweight	Significantly reducing in total fat mass, trunk fat mass, visceral fat, and load capacity index	RBR‐7jw4ry	[265]
*Lactobacillus acidophilus*, *Lactobacillus casei subsp*, *Lactobacillus lactis*, *Bifidobacterium bifidum*, *Bifidobacterium infantis*, and *Bifidobacterium longum*	Healthy	Reducing in weight, especially the visceral fat	NCT05667038	[266]
*Bifidobacterium lactis* IDCC 4301	Obesity	Decreasing body fat through changes in metabolic health parameters, such as serum triglyceride and adipokine levels	KCT0007425	[267]
*Bifidobacterium longum* and *Bifidobacterium breve*	Overweight	Decreasing abdominal visceral fat area, total abdominal fat area, and serum triglyceride levels	UMIN000047852	[268]
*Bifidobacterium animalis subsp. lactis GCL2505* and inulin	Overweight and obesity	Reducing in visceral fat	UMIN000052435	[269]
Synbiotics	MASLD	Not reduce liver fat content or markers of liver fibrosis	NCT01680640	[270]
FMT				
FMT	Healthy	Reducing in intraabdominal AT	NCT03298334	[271]
FMT	Obesity	No affecting weight loss but reducing in abdominal adiposity	ACTRN12615001351505	[272]
Bariatric surgery				
Bariatric surgery	Obesity	Recruiting active BAT, altering Akkermansiaceae, Pasteurellaceae, Carnobacteriaceae, and SCFA composition	NCT03168009	[273]
Metabolic Roux‐en‐Y gastric bypass	Type 2 diabetes	Reducing gut permeability and improving adiposity markers	NA	[274]
BAs metabolism				
UDCA	Morbid obesity	Impacting on cholesterol and BA synthesis and induces neutral lipid accumulation in both liver and visceral WAT	NCT01548079	[275]
Obeticholic acid	Familial partial lipodystrophy	Lowering hepatic TG levels	NCT02430077	[276]
Obeticholic acid	MASH	Improving NAS score	NCT02548351	[277]
EDP‐305	MASH	Reducing ALT levels and liver fat content	NCT03421431	[258, 278]
Ileo‐colonic delivery of conjugated BAs	Obesity and diabetes	Decreasing postprandial glucose, fructosamine, fasting insulin, fasting LDL, and postprandial FGF19 and increased postprandial GLP‐1 and C‐peptide	NCT02871882 NCT02033876	[121]
LPS				
IMM‐124e	MASLD	NA	NCT02316717	[279, 280]
Yaq‐001	Cirrhosis	Reducing liver injury, progression of fibrosis, and portal hypertension	NCT03202498	[247, 283]
Intestinal mucosa secretions				
Iiraglutide	Obesity	Reducing BMI	NCT04775082	[273, 282]
Semaglutide	MASH	Resulted greater weight loss	NCT04822181	[283, 284, 285]
Pemvidutide	MASLD	Significantly reducing liver fat content, hepatic inflammatory activity, and body weight	NCT05006885	[286]
Dulaglutide	Overweight/Obesity	Reducing VAT	NCT04876027	[287]
Retatrutide	Type 2 diabetes	Improving total body fat mass reduction	NCT04867785	[288]
Tirzepatide	Obesity	Reducing body weight and waist circumference	NCT05822830, NCT04660643	[288, 289]
Orforglipron	Obesity	Significantly reducing body weight	NCT05869903	[290]
Cholesin	Type 2 diabetes and Type 1 diabetes	Influencing AT	NCT03044860, NCT03093298, NCT02337660, NCT03734718	[280, 291]
Aldafermin	MASH and compensated cirrhosis	Reducing in enhanced liver fibrosis	NCT04210245	[252, 292]
Others				
Hydroxy‐methyl‐butyrate, carnosine, and magnesium	Age‐related sarcopenia	Significantly reducing in VAT and improving gut permeability	NA	[274, 284]
Equol	Healthy	Lowering odds of adiposity conditions	NCT03179657	[291]
Mild intermittent hypoxia	Overweight and obesity	Changing metabolic parameters such as adipose and peripheral insulin sensitivity	NL7120/NTR7325	[293]
Milk phospholipid‐coated lipid droplets	Formula‐fed infants	Influencing the development of the infants’ gut microbiota, their metabolomic profiles, and their body composition	NTR3683	[294]

Abbreviations: ALT, alanine aminotransferase; AT, adipose tissue; BAT, brown adipose tissue; BMI, body mass index; FGF19, fibroblast growth factor 19; FMT, fecal microbiota transplantation; GLP‐1, glucagon‐like peptide‐1; LDL, low‐density lipoprotein; LDL‐C, low‐density lipoprotein cholesterol; MASLD, metabolic dysfunction‐associated steatotic liver disease; MASH, metabolic dysfunction‐associated steatohepatitis; NA, not applicable; SCFAs, short‐chain fatty acids; UDCA, ursodeoxycholic acid; VAT, visceral adipose tissue.

### 5.1 Pharmacological Interventions

#### 5.1.1 Type 2 Diabetes

Metabolic syndrome increases the risk of developing Type 2 diabetes mellitus by up to fivefold, and the vast majority of individuals with Type 2 diabetes mellitus concurrently have metabolic syndrome [295]. Furthermore, metabolic syndrome‐related characteristics exacerbate the risk of major adverse hepatic outcomes in patients with Type 2 diabetes mellitus [296]. The Diabetes Prevention Program trial demonstrated that lifestyle intervention is remarkably efficacious in preventing the onset of diabetes mellitus [297]. Metformin may also be employed for Type 2 diabetes mellitus prevention, though its efficacy is inferior to lifestyle modification; this agent may be considered for individuals exhibiting progressive hyperglycemia despite lifestyle interventions [297]. Following a diagnosis of Type 2 diabetes mellitus, lifestyle management should be complemented by moderate‐ to high‐intensity statin therapy for LDL‐C reduction, with glycemic control targeting a hemoglobin A1c level below 7% to prevent microvascular complications [298]. Among established antihyperglycemic agents demonstrated to be beneficial in metabolic syndrome are insulin sensitizers, including metformin and thiazolidinediones (such as pioglitazone) [298, 299].

Regarding novel antidiabetic therapeutics, GLP‐1 receptor agonists [300] and sodium‐glucose cotransporter 2 (SGLT2) inhibitors [301] have been shown in multiple studies to improve cardiovascular outcomes in high cardiovascular risk populations, irrespective of Type 2 diabetes mellitus status. Additionally, novel dual GLP‐1/glucose‐dependent insulinotropic polypeptide (GIP) receptor agonists and triple (GLP‐1–GIP–glucagon) receptor agonists have proven cardiometabolic benefits among individuals with overweight or obesity complicated by diabetes mellitus [302].

The complementary mechanisms of SGLT2 inhibitors and GLP‐1 receptor agonists may further explain their broad cardiometabolic benefits in metabolic syndrome. SGLT2 inhibitors reduce renal glucose reabsorption and increase urinary glucose excretion, thereby lowering circulating glucose and insulin levels. This negative energy balance promotes a compensatory shift from carbohydrate utilization toward fatty acid oxidation and may reduce ectopic lipid accumulation in the liver and AT. In parallel, GLP‐1 receptor activation suppresses appetite through central mechanisms, delays gastric emptying, enhances glucose‐dependent insulin secretion, suppresses inappropriate glucagon release, and improves insulin sensitivity partly through weight loss and reduced lipotoxicity [303, 304]. Together, these complementary mechanisms may improve glycemic control, reduce adiposity, lower hepatic fat burden, and decrease cardiovascular and renal risk. Recent reviews suggest an expanding therapeutic role for GLP‐1‐based therapies beyond Type 2 diabetes, including obesity with cardiometabolic comorbidities, MASLD, heart failure with preserved ejection fraction (HFpEF), and pediatric obesity‐related metabolic disease [305]. With respect to safety, SGLT2 inhibitors are commonly associated with genital mycotic infections and volume depletion, whereas GLP‐1 and related receptor agonists frequently cause gastrointestinal adverse effects, including nausea, vomiting, and constipation [306].

#### 5.1.2 Hypertension

As a core diagnostic criterion of metabolic syndrome, effective management of hypertension can significantly reduce the incidence of cardiovascular events in individuals with established or at‐risk cerebrovascular disease [307]. Currently, major international academic institutions have reached consensus: for patients with elevated cardiovascular risk, blood pressure should be controlled below 130/80 mmHg, achieved through lifestyle interventions, sodium restriction, and pharmacological therapy when necessary [308]. From a dietary perspective, adoption of the Dietary Approaches to Stop Hypertension eating pattern has demonstrated definitive antihypertensive efficacy. Regarding pharmacological treatment, diuretics, calcium channel blockers, and renin–angiotensin–aldosterone system (RAAS) inhibitors confer broadly comparable cardiovascular protection in most patients with metabolic syndrome; however, RAAS inhibitors should be prioritized for those with concurrent cerebrovascular disease, diabetes mellitus with albuminuria, or heart failure—particularly when cardiac dysfunction is present—to further mitigate risks of adverse renal outcomes and cardiovascular events [309].

#### 5.1.3 Dyslipidemia

Atherosclerotic dyslipidemia presents a group of interrelated lipid metabolism disorders, specifically reflected in the increase of plasma triglyceride concentration, the decrease of high‐density lipoprotein cholesterol (HDL‐C) level, the increase of apolipoprotein B (or non‐HDL‐C) content, and the increase of the proportion of small and dense LDL particles [310]. This type of lipid abnormality is closely related to the pathophysiology of insulin resistance, and therefore presents a high detection rate in the population with metabolic syndrome [311]. Through genetic epidemiological research, it has been confirmed in academia that there is a causal relationship between elevated triglyceride levels and the onset of cerebrovascular diseases. As for the decrease in HDL‐C levels, the academic community generally regards it as an indicative marker of metabolic disorders rather than a pathological factor directly involved in disease occurrence. For hypertriglyceridemia without secondary causes, lifestyle adjustments constitute the preferred intervention for metabolic syndrome related lipid management [312]. For patients with concomitant cerebrovascular disease or cardiovascular risk stratification at medium to high levels, statins are an essential treatment option. These drugs not only exert their main lipid‐lowering effect, but also have a moderate triglyceride lowering effect. For individuals with continuously increased triglyceride level, cerebrovascular disease or diabetes and other risk factors, the combination of icosapent ethyl can be considered as an auxiliary treatment scheme, in order to obtain additional risk reduction of cerebrovascular events [313].

With regard to the relationship between high‐intensity statin treatment and abnormal glucose metabolism, existing evidence shows that this treatment mode may have a slight impact on the risk of Type 2 diabetes in individuals with prediabetes. Based on the estimated value of 1 damage event per 100 patients treated, when an individual's 10‐year risk of cerebrovascular disease exceeds the 2.5% threshold, the clinical benefits of statin treatment will significantly outweigh their potential risk of adverse reactions. Given the current lack of sufficient evidence to support net clinical benefits, aspirin is not recommended as a routine primary prophylactic medication for metabolic syndrome‐related cerebrovascular disease [313, 314]. The current guidance document of the American College of Cardiology American Heart Association proposes that low‐dose aspirin (75–100 mg, once daily orally) can be used as an alternative for primary prevention of cerebrovascular disease in a screened adult population aged 40–70 years with an increased risk of cerebrovascular disease but controllable risk of bleeding complications, but this recommendation is classified as a Class IIb recommendation [315].

#### 5.1.4 Chronic Kidney Disease

As a common complication of metabolic syndrome, the occurrence and development of chronic kidney disease reflects the superimposed damage of multiple pathological factors such as obesity, systemic inflammation, insulin resistance, Type 2 diabetes, and high blood pressure. In individuals with metabolic syndrome, the health management guidelines for cardiovascular renal metabolic syndrome clearly state that a comprehensive assessment of the risk associated with chronic kidney disease should be achieved by detecting the urinary albumin/creatinine ratio and estimating glomerular filtration rate [316]. For patients diagnosed with chronic kidney disease, it is crucial to prioritize implementing treatment strategies that delay the deterioration of kidney function. Research evidence shows that RAAS inhibitors can effectively reduce the risk of adverse renal outcomes in chronic kidney disease patients with concomitant proteinuria [317]. At the same time, SGLT2 inhibitors have demonstrated renal protective effects. These drugs can reduce adverse renal events in most patients with chronic kidney disease, and this benefit is independent of the presence of diabetes and proteinuria (NCT03594110) [318]. In addition, the new nonsteroidal mineralocorticoid receptor antagonist finerenone has been shown to reduce the incidence of adverse renal events in patients with diabetes combined with chronic kidney disease [319]. In the specific population with chronic kidney disease and Type 2 diabetes, the application of the GLP‐1 receptor agonist semaglutide can improve renal outcomes [320]. It is worth noting that the above‐mentioned renal protective drugs not only exert renal protective effects, but also have a positive impact on cardiovascular outcomes (especially the risk of heart failure), partially attributed to their effective blockade of the process of renal function decline. As the first randomized controlled study in the field of GLP‐1 receptor agonist with renal outcome as the main endpoint, the FLOW (effect of semaglutide on Type 2 diabetes with chronic renal failure) trial showed that compared with the placebo group, semaglutide significantly reduces the risk of adverse renal events and death from cardiovascular causes in patients with Type 2 diabetes with chronic kidney disease (hazard ratio 0.76, 95% confidence interval 0.66–0.88) [321]. The trial also confirmed that semaglutide treatment can reduce the risk of all‐cause mortality by up to 20% in this high‐risk population.

### 5.2 Gut–AT Axis‐Targeted Therapies

#### 5.2.1 Lifestyle Modifications

##### 5.2.1.1 Dietary Interventions

**FIGURE 5:**
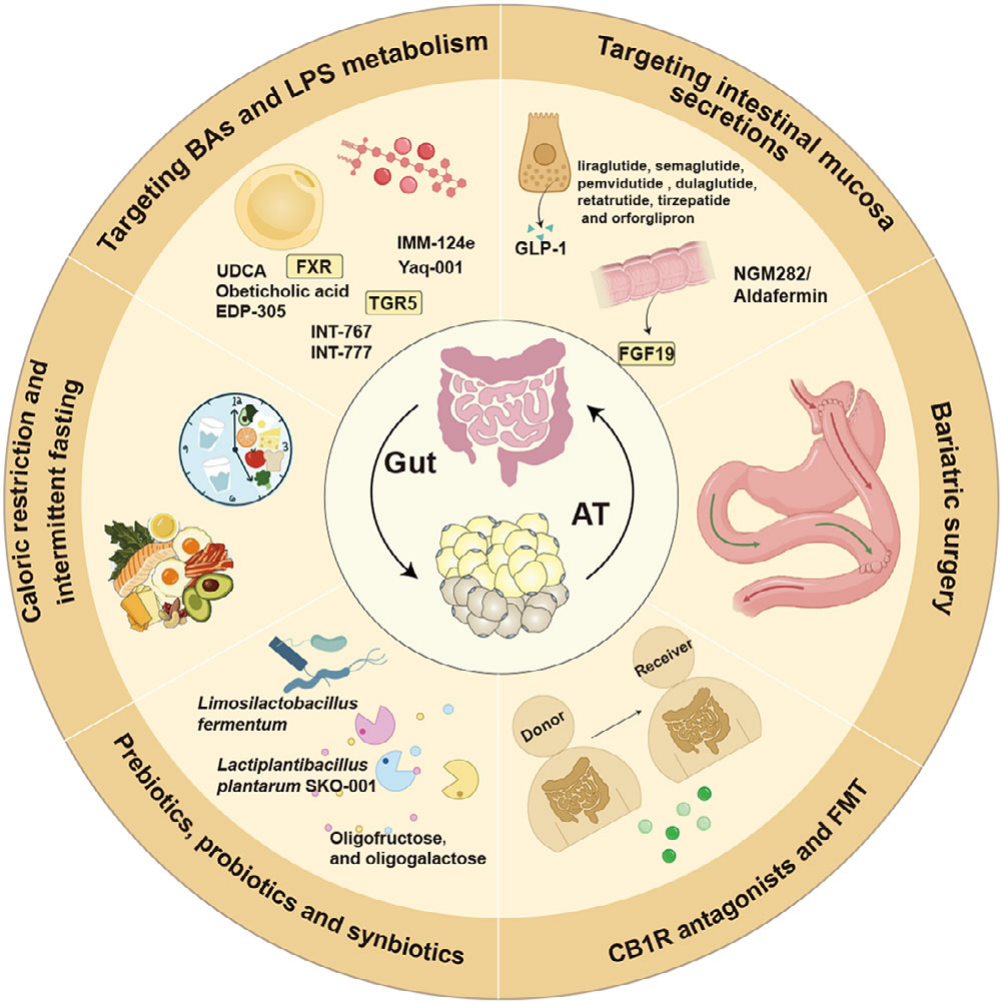
The potential therapies for the gut–adipose tissue axis in the progression of metabolic syndrome. Strategies targeting gut–AT axis range from lifestyle modifications (caloric restriction and intermittent fasting) to microbial manipulation such as targeting BAs and LPS metabolism, prebiotics, probiotics, and synbiotics, CB1R antagonist, FMT, bariatric surgery, and intestinal mucosa secretions.

Diet plays a significant role in shaping the composition, function, and diversity of the gut microbiome, particularly through caloric restriction and intermittent fasting (Figure 5) [322, 323]. For instance, a recent study investigated the crucial role of gut microbiota in the metabolic improvements induced by calorie restriction, revealing the compositional and functional changes in gut microbiota necessary for promoting WAT browning and associated metabolic enhancements [324, 325]. Notably, mice that received calorie‐restricted microbiota transplants exhibited reduced weight gain, improved glucose tolerance, and enhanced insulin sensitivity. Additionally, the expression of BAT‐specific markers in subcutaneous WAT is increased significantly, while the enhancement in visceral WAT is relatively modest [326]. Mechanistically, these effects are closely linked to the reduced expression of key bacterial enzymes required for lipid A biosynthesis, a critical component of bacterial LPS. During caloric restriction, the decrease in LPS levels mitigates the inflammatory response in WAT, thereby promoting browning and increasing insulin sensitivity [326]. However, when LPS levels rise again, the positive effects on metabolism are inhibited. In fact, depleting the LPS–TLR4 signaling pathway through genetic or pharmacological interventions can reduce WAT inflammation, promote WAT browning, and improve insulin sensitivity and hepatic steatosis [326]. In the DIRECT‐PLUS trial, following Mediterranean diet interventions, the association between fecal bile salts and body fat mass, as well as serum lipids, showed significant differences among individuals with varying quantities of gut microorganisms that carry bile salt metabolism enzymes, such as several *Ruminococcus spp* (ChiCTR2200064547) [111]. Furthermore, supplementation with pentadecanoic acid (C15:0) was able to lower LDL‐C levels and may promote an increase in the abundance of *Bifidobacterium adolescentis* (NCT05259475) [246].

Intermittent fasting is a natural and effective method for weight management, with alternate‐day being a common approach within this practice [327]. Even in a thermoneutral environment, this fasting method can selectively promote the browning of WAT without activating BAT, resulting in weight reduction, decreased liver steatosis, and improved insulin sensitivity [328]. Interestingly, similar to the effects observed in antibiotic‐induced microbiota depletion or in germ‐free mice, intermittent fasting can increase the length of the small intestine and significantly alter the composition and the content of gut microbiota. This leads to a notable increase in the Firmicutes‐to‐Bacteroidetes ratio. This alteration mirrors the changes seen in cold‐exposed mice, which are associated with enhanced glucose uptake in inguinal WAT but not in scapular BAT [329]. These findings underscore the crucial role of gut microbiota in the WAT browning induced by intermittent fasting [330]. Transplanting microbiota from intermittently fasted mice can enhance the browning characteristics of WAT and increase the length of the small intestine. However, these beneficial effects diminish when the gut microbiota is depleted. Notably, changes in gut microbiota composition resulting from intermittent fasting can lead to elevated levels of acetic acid and lactic acid in both serum and cecal contents [330]. In a randomized controlled trial, restricting food intake to the early part of the day improves fasting blood glucose levels, reduces overall body weight and fat content, alleviates inflammation, and increases gut microbiota diversity (ChiCTR2000029797). A comparison between intermittent caloric restriction and continuous caloric restriction reveals that the gut microbiota remains stable and exhibited high individual specificity (NCT02449148).

Both caloric restriction and intermittent fasting harness the gut microbiota to enhance metabolic health, primarily by reducing LPS‐induced inflammation and promoting the browning of WAT. The ability to transfer these benefits through microbiota transplantation highlights the pivotal role of gut microbes in mediating the dietary effects on AT. However, the specific microbial signatures and metabolites involved, along with the long‐term sustainability and individual variability of these dietary interventions, require further investigation.

##### 5.2.1.2 Physical Activity

Physical activity represents a cornerstone intervention for metabolic syndrome management, which has a substantial positive effect. In randomized trials of heterogeneous populations, aerobic physical activity improved each of the diagnostic components of metabolic syndrome and additional pathophysiological features, such as inflammation and endothelial dysfunction [331]. Furthermore, the addition of resistance training to aerobic activity is linked to further improvements in metabolic syndrome components and is therefore advised as part of the exercise regimen [332]. In general, moderate to vigorous physical activity on most days of the week is recommended, with a goal of 150 min or more of physical activity per week [333]. While weight loss with lifestyle modification is desirable, patients and healthcare professionals must recognize that improved dietary patterns and regular physical activity improve metabolic syndrome independent of weight loss.

#### 5.2.2 Prebiotics, Probiotics, and Synbiotics

Prebiotics, probiotics, and synbiotics offer targeted strategies to modulate gut microbiota composition and function, aiming to restore a beneficial microbial ecosystem that positively influences AT metabolism and systemic metabolic health. The term “prebiotics” refers to indigestible nutrients, including inulin‐type fructooligosaccharides, galactooligosaccharides, arabinoxylan, and arabinoxylan oligosaccharides (Figure 5) [334]. These compounds may beneficially affect gut bacteria, potentially promoting improvements in metabolic status related to AT [335]. Prebiotics can enhance the function of the intestinal mucosal barrier and stimulate the secretion of intestinal hormones that regulate appetite, maintain glucose homeostasis, and modulate inflammatory responses [336]. A recent study evaluates the role of oligofructose and oligogalactose in MASLD and finds that these two prebiotics can improve insulin resistance and reduce hyperglycemia, triglycerides, and cholesterol levels [254]. Additionally, these prebiotics significantly increase the populations of *Acidobacterium* and *Bacteroidetes*, and the acetate they produce may help repair intestinal barrier leakage caused by diet, optimize liver lipogenesis pathways, and lower levels of inflammatory markers [337]. Some novel prebiotics identified in clinical studies, such as camu‐camu, human milk oligosaccharide 2′‐fucosyllactose, dibifree, and metformin, have also preliminarily demonstrated effects in reducing body fat and lowering body weight [255, 256, 257, 258].

In addition to prebiotics, treatment with specific bacterial strains has been proposed as a therapy for metabolic syndrome (Figure 5). According to numerous in vitro and in vivo studies, probiotics, as living organisms that may positively impact human health, can improve intestinal permeability through immunomodulatory effects, competition with pathogenic microbes, and regulation of the gut–AT axis. Several clinical studies have investigated the effects of various probiotic or synbiotic interventions on body fat distribution in individuals with overweight, obesity, and related metabolic disorders. The results indicate that various strains of the genera Lactobacillus and Bifidobacterium—such as different subspecies of *Lactobacillus plantarum*, *Bifidobacterium lactis*, *Bifidobacterium longum—*either alone or in combination, can reduce indicators such as total body fat, visceral fat, subcutaneous fat area, and waist circumference to varying degrees. For example, in some clinical trials, *Limosilactobacillus fermentum* strains have been found to inhibit fasting triglycerides, increase HDL‐C, and decrease enzymatic markers of liver steatosis in patients with obesity [338]. *Lactiplantibacillus plantarum* SKO‐001 significantly decreases subcutaneous AT area, total cholesterol levels, and leptin levels in humans with obesity [262]. Additionally, *Bifidobacterium animalis* subsp. lactis GCL2505 combined with oligofructose effectively reduces visceral fat in overweight and obese individuals, while complex probiotic preparations containing multiple strains have also been shown to significantly reduce total fat mass, trunk fat, and visceral fat (UMIN000052435) [269].

Synbiotics are probiotic supplements that include prebiotic components and are emerging as a promising intervention (Figure 5). A double‐blind, randomized, placebo‐controlled Phase 2 trial evaluates the efficacy of synbiotic supplements in patients with MASLD. Although these supplements have been observed to exert a regulatory effect on gut microbiota, they do not demonstrate significant improvements in liver fat content or fibrosis severity in patients [270].

Collectively, prebiotics, probiotics, and synbiotics show potential in modulating AT distribution and metabolic parameters. Specific strains and combinations have demonstrated efficacy in reducing various fat depots and improving lipid profiles. However, their effectiveness can be strain‐specific and condition dependent, as evidenced by the limited impact of synbiotics on liver fat in patients with MASLD. Further research is necessary to identify optimal microbial consortia, elucidate mechanisms of action (e.g., barrier enhancement, metabolite production), and establish long‐term benefits across diverse metabolic conditions.

#### 5.2.3 Fecal Microbiota Transplantation

FMT is a therapeutic technique that involves transferring feces from a healthy donor into a patient's intestine (Figure 5) [339]. Currently, numerous clinical trials are exploring its potential for treating metabolic syndrome [340]. A randomized clinical trial has found that FMT can reduce liver fat accumulation by correcting gut microbiota imbalances. However, FMT appears to be more effective in “lean” individuals with fatty degeneration. Significant differences in gut microbiota composition and clinical features between the “lean” and “fat” groups suggest that the efficacy of FMT in MASLD may be limited [341]. In a double‐blind randomized trial, germ‐free mice are colonized with transitional feces from human infants who have received either vaginal or placebo inoculation. The results showed that, compared with the control group, male mice implanted with feces from infants who received vaginal inoculation exhibited a reduction in the volume of AT in their abdominal cavity. In the control group, relatively high levels of isoleucine and low levels of nucleic acid metabolites are observed, which correlated with increased intra‐abdominal AT [271]. Two clinical studies evaluated the impact of FMT on abdominal fat distribution. One trial (NCT03298334) demonstrates in healthy individuals that FMT could reduce intra‐abdominal AT. Another study (ACTRN12615001351505) shows in obese individuals that, although FMT did not significantly affect weight loss, it could reduce abdominal fat content [271, 272].

Each of these clinical studies advances our understanding of how gut microbiota dysfunction contributes to metabolic disorders and determines whether this condition can be effectively treated. It is important to emphasize that the use of FMT and bacterial strain supplementation is supported by robust preclinical data demonstrating their potential to reverse the development of metabolic diseases [342, 343].

In summary, FMT shows promise in reducing specific AT depots, such as intra‐abdominal fat, in both animal models and human studies, even in the absence of significant weight loss. However, its efficacy appears variable and may depend on the recipient's metabolic phenotype, such as lean versus obese MASLD. Preclinical evidence strongly supports its potential, but larger, well‐controlled clinical trials are essential to define its role, optimize donor selection, and establish its long‐term safety and efficacy in managing metabolic diseases.

#### 5.2.4 Bariatric Surgery

Bariatric surgery is an effective approach for helping obese patients achieve long‐term weight management, regulate gut microbiota, and improve obesity‐related complications (Figure 5) [344]. The benefits of bariatric surgery for MASLD stem not only from weight loss and fat reduction but also from decreased release of proinflammatory cytokines and reduced transport of fatty acids from AT to the liver, thereby modulating lipid and glucose metabolism. Gut‐derived peptides, such as fibroblast growth factor 15/19 (FGF15/19), may play a crucial role in promoting favorable metabolic changes following bariatric surgery [345].

Although bariatric surgery offers numerous benefits, recent evidence indicates that the gut microbiota only partially recovers postoperatively. This suggests that additional randomized controlled trials and large‐scale prospective studies are necessary to elucidate the complex relationship among gut microbiota, obesity, and weight loss surgery. Furthermore, further research is required to determine whether the regulation of gut microbiota following weight loss surgery results from anatomical, hormonal, and metabolic changes, or if it serves as a contributing factor to the surgery's beneficial effects [345]. An ongoing clinical study (NCT03168009) is investigating the impact of weight loss surgery on BAT activity and gut microbiota composition in obese patients. Preliminary findings suggest that this intervention may activate BAT and alter the abundance of bacterial families such as Akkermansiaceae, Pasteurellaceae, and Carnobacteriaceae, while modulating the metabolic profile of SCFA [273]. Additionally, research on metabolic Roux‐en‐Y gastric bypass surgery in patients with Type 2 diabetes indicates that the procedure can improve body fat distribution and related metabolic parameters by reducing intestinal permeability [274].

Bariatric surgery significantly improves metabolic health and AT function through various mechanisms, including weight loss, reduced inflammation, altered lipid flux, and regulation of gut‐derived factors such as FGF15/19. Although these changes are related to alterations in the gut microbiota, the extent to which microbial shifts are causally linked to metabolic benefits, rather than being consequences of anatomical and physiological changes, remains an active area of research. Preliminary evidence also suggests a potential impact on BAT activation, warranting further investigation.

#### 5.2.5 Targeting eCB

The eCB system, a critical component of the gut–AT axis, plays a significant role in regulating food intake, and its function may become dysregulated in obesity (Figure 5). These studies elucidate the specific molecular and cellular mechanisms of intestinal AT axis signaling mediated by the eCB system, providing potential theoretical support for developing therapeutic strategies to address human obesity and related metabolic diseases [346]. Indeed, human studies have confirmed that blood levels of eCB increase in response to a HFD and in individuals with obesity [347]. CB1R antagonists have been developed for treating human obesity. Notably, the systemic CB1R antagonist/inverse agonist rimonabant has demonstrated clinical potential by reducing weight, decreasing waist circumference, lowering circulating triglyceride levels, and increasing HDL levels. However, rimonabant is associated with psychological side effects, such as depression and anxiety, which led to its disapproval by the U.S. Food and Drug Administration for obesity treatment. This may be attributed to its ability to cross the blood‐brain barrier and interfere with cognitive function. In contrast, CB1R antagonists designed with low brain permeability may represent a safe and effective strategy for treating obesity and related metabolic disorders while maintaining antiobesity effects comparable to those of rimonabant [348].

Overall, targeting the eCB system, particularly CB1Rs, holds significant therapeutic potential for obesity and metabolic disorders. While central CB1 antagonists such as rimonabant have demonstrated efficacy, their use has been limited by psychiatric side effects. In contrast, peripherally restricted CB1 antagonists represent a promising strategy to harness metabolic benefits while minimizing adverse effects on the central nervous system.

#### 5.2.6 Targeting Microbial Metabolites

##### 5.2.6.1 Targeting BAs Metabolism

BAs and their receptors, including FXR and TGR5, are critical targets within the gut–AT axis. Agonists of these receptors have shown potential to improve metabolic health by modulating lipid and glucose metabolism, increasing energy expenditure, and enhancing AT function, although potential side effects must be considered. Ursodeoxycholic acid (UDCA), a BA derivative and FXR agonist, has been used in the treatment of leptin‐deficient obese mice (Figure 5). This study demonstrates that UDCA significantly reduces lipid droplets, decreases free fatty acids and triglycerides, improves mitochondrial function, and promotes WAT browning in ob/ob mice [349]. While UDCA shows promise as an effective treatment for metabolic syndrome, further clinical data are needed to confirm its therapeutic efficacy. In a randomized controlled pharmacodynamic study, UDCA treatment in patients with MASLD significantly affects cholesterol and BA synthesis and induces neutral lipid accumulation in both the liver and visceral WAT (NCT01548079) [275]. In MASLD, Phase II/III trials have shown that obeticholic acid and EDP‐305, both FXR agonists, decrease liver alanine aminotransferase (ALT) levels and reduce lipid accumulation [350]. However, these treatments are associated with adverse effects, including mild to severe itching, hepatotoxicity, decreased HDL‐C, and increased LDL‐C [351, 352]. Additionally, a clinical trial for familial partial lipodystrophy (NCT02430077) demonstrates that obeticholic acid effectively reduces liver TG levels [276]. In patients with metabolic dysfunction‐associated steatohepatitis (MASH), two separate studies shows that obeticholic acid (NCT02548351) improve nonalcoholic fatty liver disease (NAFLD) Activity Score [277], while EDP‐305 (NCT03421431) significantly reduces ALT levels and liver fat content [278].

Through the gut–AT axis, TGR5, another BA receptor, plays a crucial role in maintaining BA homeostasis. It is located on the membrane of L cells. LCA, deoxycholic acid, and semi‐synthetic BAs act as TGR5 agonists [353, 354]. For example, in a model of MASLD, the semi‐synthetic BAs INT‐767 and INT‐777 improve liver glucose and lipid metabolism, promote GLP‐1 production, and activate the cyclic adenosine monophosphate signaling pathway [355]. Human colonic GLP‐1‐producing EECs express TGR5. Ileo‐colonic delivery of conjugated BAs decreases postprandial glucose, fructosamine, fasting insulin, fasting LDL, and postprandial fibroblast growth factor 19 (FGF19), while increasing postprandial GLP‐1 and C‐peptide levels (NCT02871882 and NCT02033876) [356].

Targeting BA metabolism through the FXR, primarily with obeticholic acid and EDP‐305, or via TGR5 agonists such as INT‐777 and conjugated BA delivery agents, has demonstrated efficacy in improving liver function, lipid profiles, and glucose metabolism in both preclinical and clinical studies. FXR agonists show benefits in managing MASLD/MASH but are often associated with significant side effects, including pruritus and dyslipidemia. Activation of TGR5 promotes GLP‐1 secretion, thereby enhancing glucose control. Ongoing development aims to optimize therapeutic efficacy while minimizing adverse effects.

##### 5.2.6.2 Targeting LPS

Directly targeting bacterial LPS translocation or its downstream inflammatory effects represents a promising strategy to mitigate metabolic endotoxemia and its detrimental consequences on AT and the liver. IMM‐124e is an IgG‐rich extract of bovine colostrum derived from immunized cows that provides protection against LPS (Figure 5). A Phase II trial demonstrates a dose‐dependent improvement in endotoxemia and liver damage markers, notably aspartate aminotransferase, ALT, and cytokeratin‐18. However, the trial did not show a reduction in liver fat content in patients with MASLD, leading to its discontinuation (NCT02316717) [357]. Yaq‐001 is another novel agent targeting endotoxin and LPS. It is considered a new strategy to counteract gut microbiota dysbiosis and bacterial product translocation in patients with advanced liver disease, which may positively affect AT function (NCT03202498) [281, 358].

##### 5.2.6.3 Targeting Intestinal Mucosa Secretions

Harnessing the effects of gut‐derived hormones, particularly those secreted by enteroendocrine cells in response to nutrient sensing and microbial signals, offers powerful therapeutic avenues for metabolic diseases by influencing appetite, glucose homeostasis, and AT metabolism. GLP‐1 receptor agonists, such as liraglutide, semaglutide, pemvidutide, dulaglutide, retatrutide, tirzepatide, and orforglipron, have the ability to treat metabolic diseases such as obesity, MASLD, and atherosclerosis by regulating AT metabolism (Figure 5) [359]. Liraglutide interferes with the proliferation and differentiation of human adipose precursors, supporting the hypothesis of a peripheral action of GLP‐1 receptor agonists on obesity [360, 361]. Endogenous GLP‐1 is rapidly degraded by dipeptidyl peptidase‐4 within minutes. Long‐acting human GLP‐1 analogs include liraglutide and semaglutide [362]. In a Phase II clinical trial, liraglutide demonstrates increased sensitivity of the liver and systemic/local fat to insulin, resulting in decreased flow of lipophilic metabolites and inflammatory cytokines in the blood [282, 363, 364]. Semaglutide has a mechanism similar to that of liraglutide, and its effects on improving metabolism and promoting weight loss in MASLD patients are more pronounced compared with liraglutide [365]. However, it does not improve the fibrosis stage [283, 366, 367]. Furthermore, analysis of the microbiota revealed that GLP‐1 receptor agonists can alter intestinal microbiota diversity, specifically by decreasing the relative abundance of Proteus and increasing the relative abundance of *Akkermansia muciniphila*, which is associated with MASLD treatment [368]. Recently, a randomized controlled trial showed that GLP‐1 therapy increases visceral AT metabolic activity; however, this finding requires further detailed investigation [369]. In a Phase III clinical trial, orforglipron treatment leads to significantly greater reductions in body weight compared with placebo (NCT05869903). The most common adverse events with orforglipron were gastrointestinal effects, which were mostly mild to moderate [290].

After BAs activate FXR, intestinal cells in the terminal ileum release FGF19 (Figure 5). Through the portal circulation, FGF19 travels from the gut to the liver, where it binds to fibroblast growth factor receptor 4 and the coreceptor β‐klotho, leading to a decrease in BA synthesis. FGF19 analogs, including NGM282 (aldafermin), can regulate BA production, lipid metabolism, and gluconeogenesis [370]. FGF19 expression is upregulated when FXR is activated by BAs. In a Phase IIa clinical trial, NGM282 demonstrated a reduction in hepatic fat content and improvements in the NAFLD Activity Score, fibrosis score, and other liver function parameters in individuals with MASLD [371]. More recently, aldafermin has been shown to significantly reduce enhanced liver fibrosis in patients with compensated MASH cirrhosis (NCT04210245) [292].

Overall, GLP‐1 receptor agonists—primarily liraglutide, semaglutide, and orforglipron—are highly effective for weight loss and improving metabolic parameters in MASLD. Their benefits are partly mediated through direct effects on AT precursors and modulation of the gut microbiota, such as enriching Akkermansia species. Although potent, GLP‐1 therapies are associated with gastrointestinal side effects, and their effects on specific AT depots require further investigation. Additionally, FGF19 analogs like aldafermin, derived from FXR activation, show promise in reducing liver fat and fibrosis.

#### 5.2.7 Other Promising Clinical Treatments

Beyond established approaches, emerging therapies targeting the gut–AT axis include novel nutritional supplements, bioactive compounds derived from microbiota metabolism, and nonpharmacological interventions such as mild intermittent hypoxia. These therapies offer diverse mechanisms to improve metabolic health. A clinical study investigated the effects of combined supplementation with hydroxy‐methyl‐butyrate, carnosine, and magnesium in individuals with age‐related sarcopenia. The results show that this intervention significantly reduces visceral AT and improves intestinal barrier function, suggesting it may confer multiple health benefits by regulating body composition and the gut–AT axis [284]. Another clinical trial (NCT03179657) evaluates the effects of equol—an active metabolite produced by the gut microbiota from soy isoflavones—on healthy individuals and find that it is associated with a lower risk of fat‐related diseases, indicating its potential value in preventing metabolic abnormalities [291].

Mild intermittent hypoxia, an emerging metabolic intervention, has been shown to improve key metabolic parameters in overweight and obese individuals, including enhanced insulin sensitivity in AT and peripheral tissues (NL7120/NTR7325). This effect may be attributed to its ability to simulate altitude training and activate the HIF pathway, thereby promoting remodeling of energy metabolism [293].

Furthermore, a study on formula‐fed infants (NTR3683) investigated the effects of milk phospholipid‐coated lipid emulsions on early development. The results indicated that this novel formula could influence the colonization patterns of the gut microbiota, metabolomic profiles, and body composition in infants, suggesting that nutritional interventions can shape long‐term health trajectories through gut–AT interactions in early life [294].

In summary, these diverse interventions—including specific nutritional supplements (hydroxy‐methyl‐butyrate), microbiota‐derived bioactive compounds (equol), metabolic stimuli (mild intermittent hypoxia), and early‐life nutritional programming—underscore the broad potential for modulating the gut–AT axis. They provide complementary strategies to traditional therapies by targeting visceral fat reduction, improving barrier function, enhancing insulin sensitivity, and programming long‐term metabolic health, thereby expanding the arsenal against metabolic diseases.

## 6 Conclusion and Prospects

### 6.1 Conceptual Synthesis of the Gut–AT Axis in Metabolic Syndrome

Metabolic syndrome is a complex, multifactorial disorder characterized by the interplay of insulin resistance, chronic low‐grade inflammation, oxidative stress, and epigenetic modifications. These systemic mechanisms create a pathophysiological environment in which metabolic dysfunction affects multiple organs, generating self‐perpetuating cycles that accelerate disease progression. Rather than restating each downstream mechanism separately, this review positions insulin resistance, chronic inflammation, oxidative stress, and epigenetic remodeling as interconnected systemic consequences that can be amplified by persistent gut–AT axis dysregulation.

This bidirectional communication pathway involves ADEVs, adipokines, microbial metabolites, and bacterial components. ADEVs and adipokines can modulate gut microbial composition and intestinal barrier function, whereas gut microbiota‐derived metabolites—including SCFAs, LPS, BAs, eCBs, and tryptophan derivatives—reciprocally regulate AT function through receptors such as GPR41/43, TLR4, TGR5, CB1R, and AhR. These molecular interactions influence adipogenesis, lipolysis, thermogenesis, inflammatory responses, and insulin sensitivity. Therefore, dysregulation of the gut–AT axis provides a mechanistic framework for understanding the development of obesity, Type 2 diabetes, MASLD, cardiovascular complications, and other metabolic disorders.

### 6.2 Therapeutic Implications of Targeting the Gut–AT Axis

Current management of metabolic syndrome combines established pharmacological therapies with emerging interventions targeting the gut–AT axis. Conventional treatments, including insulin sensitizers, GLP‐1 receptor agonists, dual GLP‐1/GIP receptor agonists, SGLT2 inhibitors, RAAS inhibitors, statins, and agents such as icosapent ethyl, remain essential for controlling hyperglycemia, obesity, hypertension, dyslipidemia, renal risk, and cardiovascular complications. Although these agents primarily address downstream systemic manifestations, their interactions with gut microbiota composition, microbial metabolites, intestinal barrier integrity, and adipose inflammation remain underexplored and may partly explain interindividual differences in therapeutic response.

Gut–AT axis‐targeted strategies provide additional therapeutic opportunities. Lifestyle interventions, including Mediterranean dietary patterns and intermittent fasting, may improve metabolic parameters by modulating microbial diversity and metabolite production. Prebiotics, probiotics, synbiotics, and specific microbial candidates such as *Akkermansia muciniphila* and *Bifidobacterium spp*. aim to restore beneficial microbial populations and metabolic functions. Bariatric surgery may improve metabolic health partly through changes in SCFA levels, BA signaling, gut hormone secretion, and BAT activity. Pharmacological approaches targeting FXR, TGR5, peripheral CB1R, and metabolic endotoxemia also show promise. However, most current evidence remains associative, and stronger causal validation is required before these strategies can be broadly translated into clinical practice.

### 6.3 Key Unresolved Scientific Questions

Despite substantial progress, several key scientific questions remain unresolved. First, it remains difficult to determine whether specific gut microbiota changes and microbial metabolites are causal drivers of metabolic syndrome or secondary markers of disease progression. Many studies still rely on cross‐sectional associations, and some mechanisms are inferred without direct causal validation. Future research should combine Mendelian randomization, longitudinal cohorts, controlled interventions, metagenomics, metabolomics, lipidomics, and tissue‐specific experimental models to clarify causal relationships.

Second, the tissue‐specific and species‐specific effects of gut‐derived signals on AT remain incompletely understood. Single‐cell and spatial omics technologies have revealed depot‐specific responses, including differences between visceral and subcutaneous WAT and between WAT and BAT [372, 373]. However, findings from germ‐free mice, HFD‐fed rodents, and in vitro metabolite stimulation models do not always reproduce human physiology. For example, SCFAs may promote WAT browning and thermogenesis more strongly in mice than in humans, reflecting species‐specific differences in BAT activity and metabolic regulation. Similarly, succinate may exert context‐dependent or even paradoxical effects through GPR91 depending on dose, tissue context, and disease state [374, 375].

Third, the biological significance of adipose‐associated microbial signals requires further clarification. Animal experiments on gut–AT axis are crucial for mechanistic studies. However, their translatability to humans is limited. For example, SCFAs promote WAT browning and thermogenesis more effectively in mice than in humans, reflecting species‐specific differences in BAT activity and metabolism. Germ‐free or HFD mouse models reveal links between microbiota dysbiosis and metabolic dysfunction but inadequately replicate the dietary, genetic, and environmental complexity of humans. Technically, metagenomic sequencing cannot distinguish between live and dead bacterial DNA, which biases assessments of microbial activity, and fecal samples fail to capture microbial dynamics in critical regions such as the small intestine. Although spatial transcriptomics and single‐cell technologies provide tissue‐specific insights, they remain costly and technically challenging. Most studies are correlational—for example, the association between *Oscillibacter valericigenes* and WAT inflammation—without causal validation [376]. Additionally, metabolite effects, such as succinate acting via GPR91, are often tested at nonphysiological doses in vitro, limiting their biological relevance. To advance the field, research should integrate organoid cocultures, longitudinal interventions, multiomics approaches, and robust statistical frameworks to strengthen causal inference and move beyond associations toward mechanistic understanding.

### 6.4 Technological Challenges and Future Directions

Recent technological advances have illuminated the complexity of this axis while revealing significant challenges. Single‐cell and spatial omics technologies have uncovered depot‐specific responses (visceral vs. subcutaneous, WAT vs. BAT) to microbial signals [372, 373]. For example, integrated multiomics data demonstrate that tetrahydroxanthohumol primarily reduces the abundance of proinflammatory gut microbes, such as *Oscillibacter valericigenes*, which can promote macrophage‐associated inflammation in WAT [376]. These findings suggest that multiomics approaches may help identify microbial signatures and metabolites that are functionally linked to AT remodeling.

Nevertheless, several methodological and translational challenges remain. Many associations between gut microbiota, microbial metabolites, and metabolic outcomes lack causal validation. Conflicting evidence also exists for several metabolites. SCFAs may exert beneficial metabolic effects in some contexts but promote lipogenesis in others, whereas the role of TMAO remains controversial across cohorts and disease states. Emerging evidence suggests that gut microbiota dysbiosis and related metabolites, including SCFAs, TMAO, and BAs, are associated with AT dysfunction and cardiovascular disease progression, providing a new perspective on the gut–AT axis in cardiovascular health [377, 378, 379]. However, further animal and human studies are needed to determine whether these associations reflect causal mechanisms.

In this broader context, the gut–AT axis should be integrated into a gut–AT–liver–cardiovascular network. Recent state‐of‐the‐art reviews emphasize that MASLD is not only a hepatic manifestation of metabolic syndrome but also a multisystem cardiometabolic disorder closely associated with atherosclerotic cardiovascular disease, vascular inflammation, myocardial remodeling, and adverse cardiovascular outcomes [380]. Mechanistically, gut dysbiosis and intestinal barrier dysfunction may promote microbial product translocation and systemic inflammation, whereas dysfunctional visceral AT increases free fatty acid flux, adipokine imbalance, macrophage‐associated inflammation, and insulin resistance. These signals converge on the liver to aggravate steatosis, lipotoxicity, and MASLD progression, particularly in individuals with Type 2 diabetes [303]. The same metabolic‐inflammatory network may predispose patients to HFpEF. MASLD and obesity share common drivers of HFpEF, including insulin resistance, systemic inflammation, endothelial dysfunction, myocardial stiffness, and impaired cardiometabolic substrate utilization [305, 381]. Importantly, this interaction may begin early in life: childhood obesity‐related MASLD can increase the risk of youth‐onset Type 2 diabetes and may accelerate long‐term cardiometabolic risk trajectories [382]. These recent findings support an expanded framework in which gut–AT dysfunction, hepatic steatosis, Type 2 diabetes, and cardiovascular disease are interconnected phenotypic presentations of metabolic syndrome rather than isolated complications.

Future studies should prioritize hypothesis‐driven experimental designs with adequate statistical power, standardized microbiome profiling, inclusion of diverse populations, and systematic reporting of negative findings. CRISPR–Cas‐based microbial gene editing, organoid cocultures, tissue‐specific knockout models, single‐cell transcriptomics, and spatial metabolomics may help dissect host–microbe–metabolite–receptor interactions. Ultimately, integrating gut microbiota signatures, host genetics, metabolic phenotypes, lifestyle factors, and multiomics biomarkers will be essential for developing precision strategies targeting the gut–AT axis.

In conclusion, the gut–AT axis represents a dynamic and multifactorial hub in which systemic mechanisms of metabolic syndrome converge. By elucidating the molecular interplay among insulin resistance, inflammation, oxidative stress, epigenetic programming, gut microbiota, and AT dysfunction, future research may support the development of precision‐based therapies that combine conventional pharmacological treatment with microbiome‐based and adipose‐focused interventions to restore metabolic homeostasis and prevent complications of metabolic syndrome.

## Author Contributions

Mengyao Yan performed reference searches, wrote the manuscript, and created the tables and figures. Qian Xiang conceptualized, reviewed, and edited the manuscript. The final version of the work has been read and approved by all authors.

## Funding

This study was supported by grants from the National High Level Hospital Clinical Research Fund (Peking University First Hospital Interdisciplinary Research Project, 2023IR06) and National Natural Science Foundation of China (no. 82373950, 82274024, 82404749, 82404751, and 82300799). This article mainly used PubMed and Endnote X9 for reference collection.

## Ethics Statement

The authors have nothing to report.

## Conflicts of Interest

The authors declare no conflicts of interest.

## Data Availability

The authors have nothing to report.
